# Influence of Native Defects on the Structural, Electronic, Thermal, and Ionic Transport Properties of YBO_3 ± δ_ Perovskites

**DOI:** 10.1021/acs.inorgchem.5c03650

**Published:** 2026-01-23

**Authors:** Nathan Rabelo Martins, Alan Antônio Das Graças Santos, Luiz Augusto Ferreira de Campos Viana, Luisa Scolfaro, Daiane Damasceno Borges, Pablo Damasceno Borges

**Affiliations:** † Instituto de Ciências Exatas e Tecnológicas, 28120 Universidade Federal de Viçosa, Campus Rio Paranaíba, Rodovia MG 230, km 7, Rio Paranaíba, Minas Gerais 38810-000, Brazil; ‡ Instituto de Física, 295760 Universidade Federal de Uberlândia, Av. João Naves de Ávila 2121, Campus Santa Mônica, Uberlândia, Minas Gerais 38400-902, Brazil; § Department of Physics, 186065 Texas State University, San Marcos, Texas 78666, United States; ∥ Instituto Federal de Educação, Ciência e Tecnologia de Minas Gerais (IFMG) - Campus Avançado Arcos, Av. Juscelino Kubitschek 485, Arcos, Minas Gerais 35588-000, Brazil; ⊥ Department of Materials Science, Military Institute of Engineering (IME), Praça General Tibúrcio 80, Urca, Rio de Janeiro, Rio de Janeiro 22290-270, Brazil

## Abstract

The development of cathode materials with high ionic conductivity and thermomechanical compatibility remains a major challenge for advancing solid oxide fuel cells (SOFCs), especially at intermediate operating temperatures. In this work, we present a comprehensive computational study of orthorhombic YBO_3±δ_ perovskites, where B = Sc, Ti, V, Cr, Mn, Fe, Co, or Ni, using a combined approach of density functional theory (DFT) and molecular dynamics (MD). We evaluate the influence of native defects, oxygen vacancies (V_O_), and interstitial oxygen (O_i_) on the structural, electronic, thermal, and ionic transport properties. Our results show that YCrO_3_ and YTiO_3_ exhibit thermal expansion coefficients (TECs) compatible with widely used electrolytes such as YSZ. Hybrid DFT calculations reveal that pristine compounds, except for YNiO_3_, behave as moderate-to-wide bandgap semiconductors, with native defects generally reducing the bandgap. MD simulations indicate that pristine (Pr) materials show negligible oxygen ion mobility, while the presence of defects substantially enhances ionic conductivity. O_i_ defects are particularly effective, yielding lower activation energies and higher self-diffusion coefficients compared to V_O_. These findings demonstrate the critical role of defect engineering and highlight the potential of combined MD and DFT methodologies for the analysis and design of SOFC cathodes.

## Graphical abstract

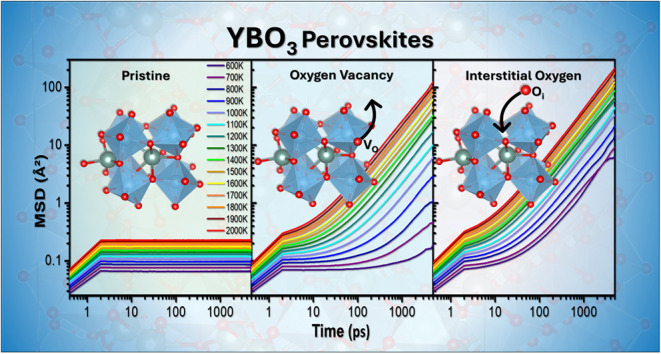

## Introduction

The growing environmental concerns associated with fossil fuel consumption has intensified the search for sustainable and renewable energy alternatives. The combustion of these fuels generates toxic gases such as carbon monoxide and greenhouse gases like carbon dioxide, which are major contributors to global warming. Furthermore, the projected reduction of fossil fuel reserves within the next few decades underscores the urgent need for cleaner energy sources.^1,2^

Solid oxide fuel cells (SOFCs) have emerged as promising devices in this context, as they convert the chemical energy of a fuel directly into electrical energy through redox reactions. Depending on the fuel used, their only byproduct may be water vapor, thus significantly reducing environmental impact.^3,4^ SOFCs are generally classified based on their electrolyte material, efficiency, and operating temperature range. Among these, oxygen ion-conducting SOFCs offer high power densities but demand high operating temperatures, typically higher than 1000 K, which impose limitations related to material degradation, sealing issues, and restricted application flexibility.^5,6^

To reduce these temperatures while maintaining high electrochemical performance, the development of advanced cathode materials is critical. These materials must exhibit high oxygen reduction reaction activity, enhance oxygen ion migration, and be chemically and thermomechanically compatible with other SOFC components, especially the electrolyte. Essential parameters to consider include thermal expansion coefficient (TEC), chemical stability, and interfacial reactivity between cathode and electrolyte.^5–7^

Yttria-stabilized zirconia (YSZ) remains the most widely used electrolyte in SOFCs,^8^ while Ni-YSZ is the preferred anode due to its excellent electronic conductivity and compatible TEC.^9^ As for cathodes, perovskite-type oxides such as La_1–*x*_Sr_*x*_MnO_3_ (LSM), La_1–*x*_Sr_*x*_CoO_3_ (LSC), and La_1–*x*_Sr_*x*_Co_1–*y*_Fe_*y*_O_3_ (LSCF) have received considerable attention. However, these materials generally require temperatures above 1000 K to achieve adequate ionic conductivity and may exhibit poor chemical stability in reactive environments.^3,5,6,10,11^

Given these limitations, another perovskite compounds are being explored to improve ionic conductivity and stability at intermediate temperatures (700–900 K). Perovskites have the general formula ABO_3_, where A-site cations are typically rare-earth or alkaline elements, and B-site cations are transition metals. These materials can crystallize in various symmetries, like orthorhombic, rhombohedral, cubic, and tetragonal.^12^

Native defects such as oxygen vacancies (V_O_) and interstitial oxygen atoms (O_i_) are commonly observed in perovskite oxides due to the inherent flexibility of their crystal structures. The perovskite lattice offers energetically favorable sites for the formation and accommodation of such defects, which can be further promoted by external factors such as high temperatures and reactive atmospheres.^10,13^ These native defects can significantly influence the physical and chemical behavior of perovskites, particularly affecting their electronic and ionic transport properties features that are critical for SOFC applications.^11,14^

While the contribution of V_O_ to oxygen ion diffusion is well established, the influence of O_i_ remains relatively underexplored and may provide alternative or synergistic mechanisms for ionic conduction, particularly at reduced temperatures. Recent studies have demonstrated that interstitial defects can notably alter the electronic structure and bandgap, enhancing charge transport properties in perovskite systems.^15–17^

The use of computational approaches, such as classical molecular dynamics (MD) and density functional theory (DFT) has been essential for exploring the properties of such materials. From MD simulations, ionic and thermal mobility are studied over time, while the electronic structure and energetic properties of defects are obtained from DFT calculations. When used synergistically, these methods offer a powerful approach for understanding and predicting material performance under realistic operating conditions.^14–16,18–20^

In this work, we present a systematic investigation of orthorhombic perovskites of the form YBO_3±δ_ (B = Sc, Ti, V, Cr, Mn, Fe, Co, Ni), focusing on the influence of native defects, both V_O_ and O_i_, on their structural, electronic, thermal, and ionic diffusion properties. The selection of B-site cations was based on previous screening studies for SOFCs applications.^6^ Our results, obtained through combined MD and DFT simulations, reveal that defect engineering can significantly enhance oxygen mobility while preserving thermomechanical compatibility with typical SOFC electrolytes such as YSZ. Furthermore, we discuss how specific defect mechanisms impact the band structure and activation energies for oxygen diffusion, providing guidelines to better understand the effect of native defects in different perovskites.

## Computational Methods

Ab initio calculations based on DFT were performed using the Vienna Ab Initio Simulation Package (VASP, version 6.2.0).^21–24^ The generalized gradient approximation (GGA) with the Perdew–Burke–Ernzerhof (PBE) functional was applied for structural relaxations.^25^ HSE06 functional was used to accurately predict the electronic density of states and bandgap values.^26,27^ The projector augmented-wave (PAW) pseudopotentials adopted in this work are listed in Table S1, and a plane-wave cutoff energy of 450 eV was used, and no electronic smearing was used in any of the DFT simulations.

The orthorhombic (*Pbnm*, #62) and cubic (*Pm-3m*, #221) phases of YBO_3_ were considered. The primitive orthorhombic unit cell contains 20 atoms (4 Y, 4 B, 12 O), while the cubic cell comprises 5 atoms. Structural relaxations were converged until residual forces were below 10 meV/Å. For the structural optimizations, only the primitive unit cell was used, together with a 4 × 4 × 3 k-point mesh. For the density of states (DOS) analysis, a 2 × 2 × 1 orthorhombic supercell containing 80 atoms was employed, along with a 2 × 2 × 3 k-point mesh.

Classical MD simulations were conducted using the LAMMPS, version 20240207, code.^28,29^ A 4 × 4 × 3 supercell of the orthorhombic structure (960 atoms) and a 5 × 5 × 5 supercell of the cubic structure (625 atoms) was used, see Figure S1. The systems were equilibrated in three steps: microcanonical (NVE), canonical (NVT), and isothermal–isobaric (NPT) ensembles, each lasting 100 ps with a 1 fs time step. The Nosé–Hoover thermostat and barostat were applied during NPT isotropic simulations. All MD simulations were primarily performed using an isotropic NpT ensemble to preserve the experimental lattice symmetry during finite-temperature simulations. Since validation tests using an anisotropic NpT ensemble confirmed fully anisotropic relaxation led to deviations in lattice-parameter ratios relative to experimental values, reflecting known limitations of classical fixed-charge force fields. Therefore, isotropic NpT was adopted to avoid lattice parameters distortions while maintaining a geometrically realistic diffusion environment.

Native defect simulations (V_O_ and O_i_) were introduced only in the orthorhombic cell preserving charge neutrality in all cases, in both MD and DFT calculations. Finite-size effects were evaluated by explicit size-convergence tests and found to be negligible under the present conditions.^30^

MD simulations were performed with a low native defect concentration of 0.52%, appropriate for preserving computational efficiency while capturing defect dynamics. Since MD simulations are less sensitive to initial atomic configurations, the positions of V_O_ and O_i_ were randomly assigned at the start of the simulations. In the employed MD model, oxygen ions carry a formal charge of 2–, and the insertion or removal of oxygen atoms alters the total charge of the system. To maintain charge neutrality, the formal charges of all B site cations were uniformly adjusted to compensate for the net charge introduced by the native defects. This charge compensation approach is consistent with the methodology proposed by Kaub et al. and is particularly suited to modeling systems with low defect concentrations.^31^ Although different defect concentrations were used in DFT and MD due to methodological constraints, this variation did not significantly influence the structural trends and qualitative insights obtained from both approaches as shown in previous work.^15^

Long-range electrostatic interactions were treated with the Ewald summation method, and atomic trajectories were integrated using the Verlet algorithm.^32^ Short-range interactions were described using the Buckingham potential within the Born model, as shown in eq 1, where *r* denotes the distance between ions *i* and *j*, and *A*, *ρ,* and *C* are fitted empirical parameters.^33–35^

1 $$V_{i,j}\left(r\right)=A\exp\left(\frac{-r}{\rho }\right)-\frac{C}{r^{6}}$$

To account for the electronic polarizability of O^2–^ ions, the core–shell model was employed, where each ion is divided into a massive core and a massless shell, with charge (*Y*), connected by a harmonic spring (*k*), as defined in eq 2:

2 $$\alpha =\frac{Y²}{k}$$

In this study, short-range interatomic interactions were modeled using Buckingham potential, which requires the parametrization of repulsive and attractive terms for each ionic pair. The parameters for Y^3+^–O^2–^ and O^2–^–O^2–^ interactions were adapted from Dutra et al.,^36^ while those for B^3+^–O^2–^ were obtained through a parameter optimization procedure using the General Utility Lattice Program (GULP).^37–40^ The fitting aimed to reproduce key structural properties of reference materials, including lattice parameters, cell volume, and atomic positions. Reference data were sourced from the Materials Project database,^41^ which provides *ab initio*-derived crystallographic information.

To validate the robustness of the B^3+^-O^2–^ parameters, we performed additional tests considering only the YTiO_3_ structure, in which three independent test configurations were investigated, each employing Ti_2_O_3_ combined with different structural forms of YTiO_3_: orthorhombic, cubic, and a mixed orthorhombic and cubic set. Although slight variations in the fitted parameters, particularly the repulsive *A* factor were observed, all configurations produced similar optimized volume and bulk modulus curve (see Figure S2), suggesting internal consistency in the parametrization. Given the availability of experimental data, the orthorhombic phase of YBO_3_ and the structure of B_2_O_3_ were selected as the standard models for final fitting. For systems involving Co^3+^-O^2–^ and Ni^3+^-O^2–^ interactions, convergence could not be achieved using Co_2_O_3_ and Ni_2_O_3_ oxides, and thus only the orthorhombic YBO_3_ structure was employed in these cases. The complete set of Buckingham parameters used in the simulations is presented in Table 1.

**Table 1:** Interatomic Potential Parameters Used in MD Simulations of YBO_3_ Perovskites^a^

**Short range interaction**				**Core shell model**		
**Interaction**	***A*** **(eV)**	***ρ*** **(Å)**	***C*** **(eV·Å** ^**6**^)		***Y*** (e)	***k*** **(eV·Å** ^**–2**^)
**Y** ^**3+**^ **–O** ^**2–**^	1345.10	0.3491	0.0	-	-	-
**Sc** ^**3+**^ **–O** ^**2–**^	1097.00	0.3414	0.0	-	-	-
**Ti** ^**3+**^ **–O** ^**2–**^	570.69	0.3822	0.0	-	-	-
**V** ^**3+**^ **–O** ^**2–**^	10002.54	0.2375	0.0	-	-	-
**Cr** ^**3+**^ **–O** ^**2–**^	525.70	0.3846	0.0	-	-	-
**Mn** ^**3+**^ **–O** ^**2–**^	1447.72	0.3163	0.0	-	-	-
**Fe** ^**3+**^ **–O** ^**2–**^	1438.60	0.3148	0.0	-	-	-
**Co** ^**3+**^ **–O** ^**2–**^	3481.23	0.2600	0.0	-	-	-
**Ni** ^**3+**^ **–O** ^**2–**^	3877.53	0.2581	0.0	-	-	-
**O** ^**2‑**^ **–O** ^**2–**^	22764.00	0.1490	27.88	**O** ^**2–**^	–2.240	65.30

a The radial cut-off distance was set to 12 Å.

The TEC was calculated from the linear fit of volume vs temperature data obtained from isotropic NPT simulations in the range of 600–1300 K, and oxygen ion diffusion was evaluated by computing the mean squared displacement (MSD) as a function of time, according to eq 3 and the self-diffusion coefficient *D_C_* was obtained using the Einstein relation (eq 4), where ⟨*r* ^2^(*t*)⟩ represents the time dependent MSD, *t* is time, *N* is the number of particles and ***r*** is the position vector.

3 $$\left\langle r^{2}\left(t\right)\right\rangle =\frac{1}{N}\overset{N}{\underset{i=1}{\sum }}\left|r_{i}\left(t\right)-r_{i}\left(0\right)\right|²$$

4 $$\left\langle r^{2}\left(t\right)\right\rangle =2dD_{C}t$$

Activation energies *E_a_* were extracted from Arrhenius fits of *D_C_* vs 1/*T* according to eq 5, where *D*_0_ is a temperature independent pre-exponential factor, *k_B_* is the Boltzmann constant, and *T* is the absolute temperature.

5 $$D_{C}=D_{0}\;\exp\left(\frac{-E_{a}}{k_{B}T}\right)↔\ln D_{C}=\ln D_{0}-\frac{E_{a}}{k_{B}T}$$

## Results

### Structural Properties

To compare the energetic stability of different YBO_3_ crystalline phases, we systematically investigated their structural properties through density functional theory (DFT) and molecular dynamics (MD) simulations. All YBO_3_ compounds were studied in both their cubic and orthorhombic crystal forms. In the DFT calculations, we varied the unit cell volume and performed full relaxations of both the ionic positions and electronic structure at each volume point. For the classical approach, a similar procedure was adopted, in which only atomic positions were relaxed using the FIRE (Fast Inertial Relaxation Engine) algorithm implemented in the LAMMPS code,^42^ within the classical force-field approximation. No time evolution or thermostat was performed, and thus no molecular dynamics simulations in the strict sense were carried out at this stage. Instead, these calculations correspond to static structural relaxations at zero Kelvin, analogous to the DFT calculations but based on the empirical Buckingham potential.

The equilibrium volume was identified as the configuration corresponding to the minimum total energy. Figure 1a and b show the total energy versus volume curve for YScO_3_, obtained from DFT and MD simulations, respectively. In both cases, the orthorhombic phase is energetically more favorable than the cubic phase. Similar results were verified for other systems, where YBO_3_ (B = Ti, V, Cr, Mn, Fe, Co, Ni), where orthorhombic structure is the most stable configuration for all considered B site cations, in agreement with experimental findings.^43–50^ Figure 2 presents an overview of the calculated volume values as a function of the element occupying the B site, only for orthorhombic structures. In addition to the close agreement between methods, the results reveal a clear trend, the crystal volume systematically decreases with the decreasing ionic radius of the B site cation. The largest deviation in the optimized volume, relative to experimental values shown in Figure 2, was found to be 2.28% for B = Sc in the DFT calculations and 4.15% for B = V in the MD simulations. Regarding the lattice parameters, the error remained below 2% for both computational approaches.

**Figure 1:**
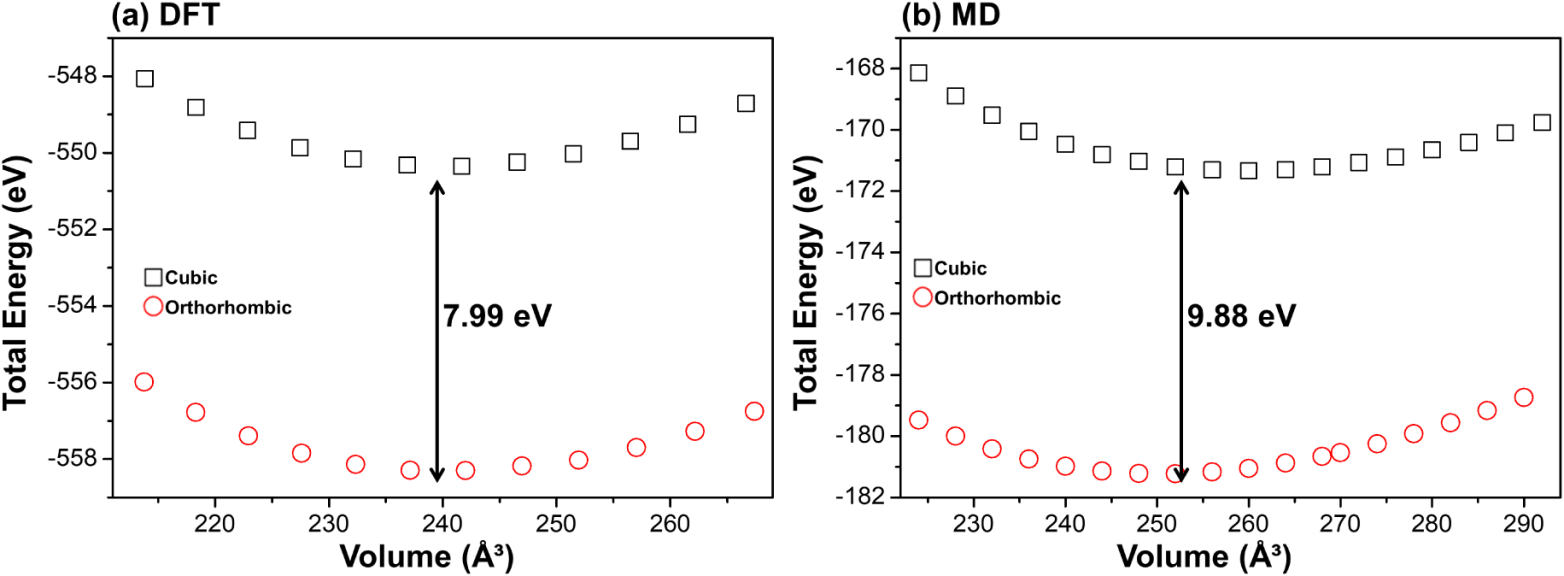
Total energy (eV) versus volume (Å^3^) referring to a cell with 4 formula units of YScO_3_, corresponding to the orthorhombic unit cell, obtained from (a) DFT (GGA-PBE) calculations and (b) MD simulations for both cubic and orthorhombic structures.

**Figure 2:**
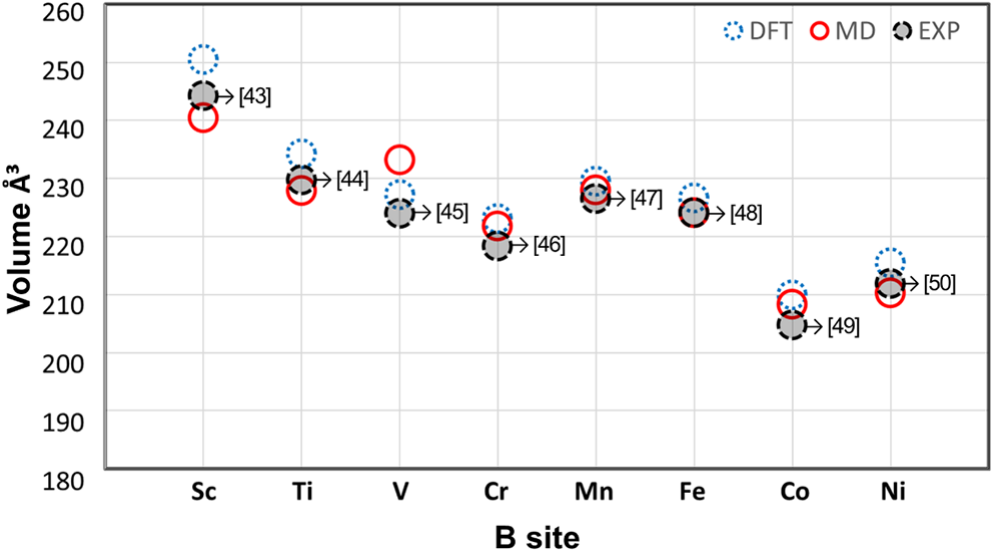
Comparison of optimized volumes per orthorhombic unit cell as a function of the B-site element, based on DFT and MD calculations, for orthorhombic YBO_3_ structures. Available experimental data are shown by shaded circles.^43–50^

Additional analyses into the structural behavior of YBO_3_ compounds are provided in the Supporting Information. Figure S3 displays the total energy curves as a function of volume for each B-site cation, obtained through both DFT and MD calculations. The parabolic character shows the existence of a well-defined energy minimum for all considered cations, indicating the mechanical stability of the optimized orthorhombic structures. Furthermore, the behavior of the curve and the proximity between the MD and DFT minimum highlights the consistency between both methodologies.

Table S2 further complements this analysis, showing that not only do the calculated volumes agree well between the methods, but also the lattice constants, bulk modulus, and interatomic distances for each compound. The computed bulk modulus is on the order of 10^2^ GPa for all materials, with the MD values consistently slightly higher than the DFT results. The findings indicate that both MD and DFT methods are effective in describing the structural properties of YBO_3_-type compounds, exhibiting strong agreement not only with each other but also with experimental and theoretical data available in the literature.

All orthorhombic YBO_3_ perovskites exhibit a characteristic distortion of the BO_6_ octahedra (Figure 3a), as evidenced by deviations from the ideal B–O–B bond angle of 180°, which would be expected in a perfectly undistorted *Pbnm* or cubic perovskite lattice. This distortion arises from the tilting and rotation of the octahedra, commonly observed in orthorhombic perovskites of the space group *Pbnm*.^12,51^

**Figure 3:**
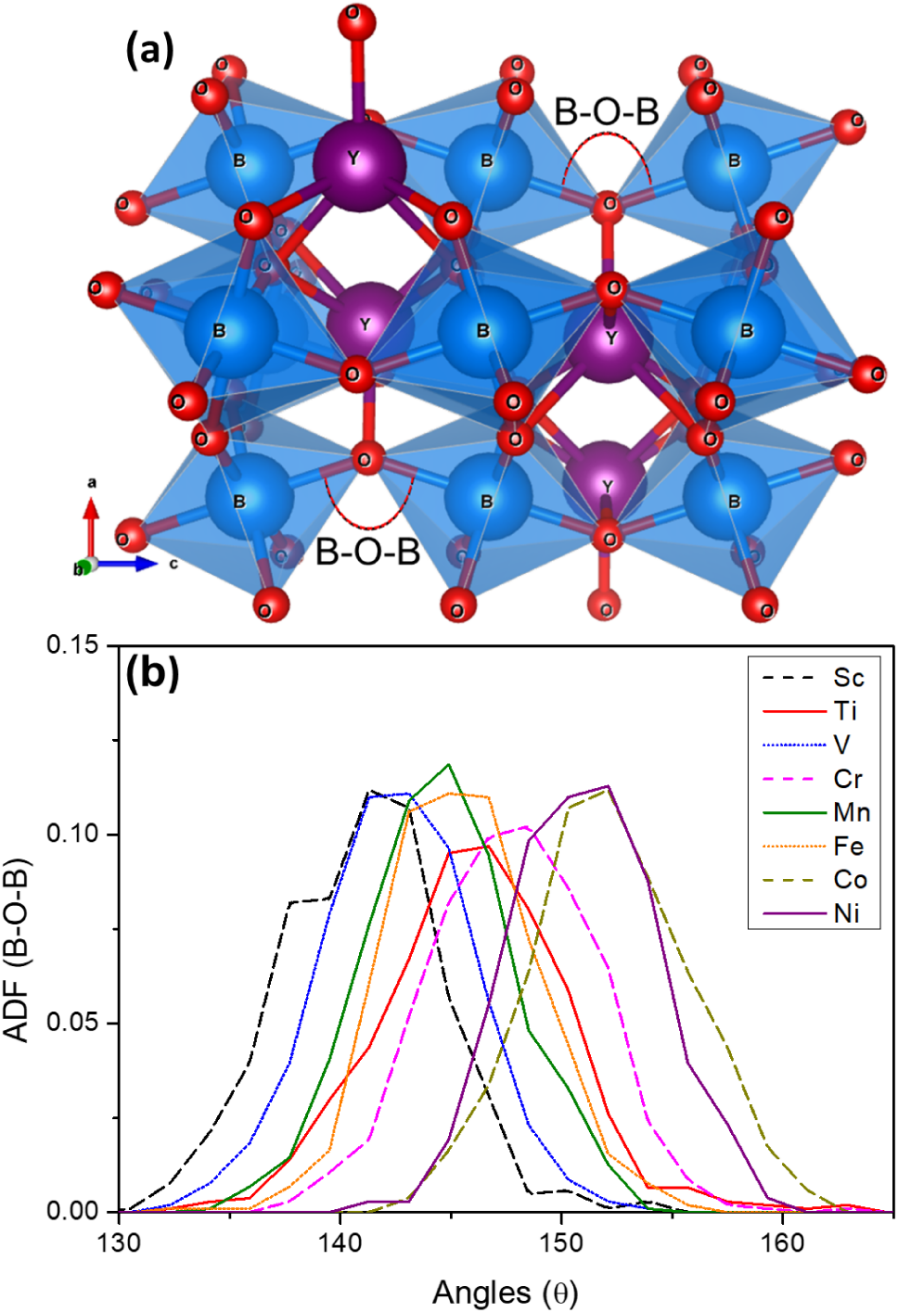
(a) Crystal structure of orthorhombic YBO_3_ highlighting the B–O–B bond angle, which reflects the distortion of the BO_6_ octahedra. (b) Distribution of B–O–B angles for different B elements (B = Sc, Ti, V, Cr, Mn, Fe, Co, and Ni), obtained from MD simulations at 300 K.

To quantify this structural feature, we computed the angular distribution function (ADF) of the B–O–B angles from MD simulations at 300 K. As shown in Figure 3b, all systems exhibit broad distributions of B–O–B angles, ranging predominantly from 130° to 160°. The ADF peaks occur around 142° for B = Sc and V, indicating moderate tilting, whereas for B = Co and Ni, the distributions shift toward higher angles (∼155°), suggesting comparatively less distortion.

The magnetic stability was investigated through spin-polarized GGA-PBE DFT calculations to determine the magnetic moments of YBO_3_ perovskites. To identify the most stable magnetic configuration, three different antiferromagnetic (AFM) arrangements (A-type, B-type, and C-type) were considered as illustrated in Figure S4. Table S3 summarizes the valence electron configurations, calculated partial magnetic moments, and magnetic ordering as a function of the B-site cation in the ground state for orthorhombic YBO_3_. In these compounds, the B-site cations adopt an oxidation state of +3, consistent with charge neutrality in the perovskite lattice composed of Y^3+^ and O^2–^ ions. For Ti^3+^, V^3+^, and Ni^3+^ on site B, the compounds exhibit ferromagnetic (FM) ordering, while Cr^3+^, Mn^3+^ and Fe^3+^ show antiferromagnetic (AFM) nature. Sc^3+^ and Co^3+^ are found to be diamagnetic (DM), with zero net magnetic moment.

It is noteworthy that for B = Ti, V, Cr, Mn, Fe, and Ni, the energy difference between FM and AFM states is relatively small, lower than 0.3 eV (see Table S3), suggesting that magnetic phase transitions may be induced under external perturbations such as strain, pressure, temperature, or chemical doping. This behavior agrees with previous observations in related perovskite systems, including LaBO_3_ and YBO_3_, where the electronic structure is modified due to crystal and exchange fields effects.^51–56^

### Electronic Properties

The electronic properties of YBO_3±δ_ perovskites were investigated through the calculation of the total (TDOS) and partial (PDOS) density of states, considering the orthorhombic structures. In such cases, although experimental studies confirm the semiconducting character of YBO_3_ compounds, conventional electronic structure calculations using the GGA-PBE functional tend to incorrectly predict a conductor character. This well-known limitation arises because GGA-PBE fails to accurately describe the strong electronic correlations in the system.^15,57–59^ To overcome this issue and achieve an accurate description of the electronic structure, particularly the band gap and the alignment of the valence and conduction bands, the HSE06 hybrid functional was used.

The electronic structure is similar across all the investigated perovskites YBO_3_. In the pristine systems, the valence band is primarily characterized by strong hybridization between O­(p) and B­(d) orbitals. The contribution from the B­(d) orbitals to the valence band increases progressively with d orbital occupancy; for instance, perovskites containing Ni display a significantly higher density of d states in the valence region than those containing Sc. The bottom of the conduction band is primarily composed of unoccupied B­(d) states, which then hybridize with Y­(d) states at higher energy levels. The YCrO_3_ system, shown in Figure 4a–i and b–i, is a representative case. For the Pr-AFM structure, which corresponds to the most favorable magnetic configuration in the ground state, the system exhibits a strong hybridization between O­(p) and Cr­(d) orbitals which forms the top of the valence band, while the bottom of the conduction band consists of Cr­(d) levels that hybridize with Y­(d) levels at higher energies.

**Figure 4:**
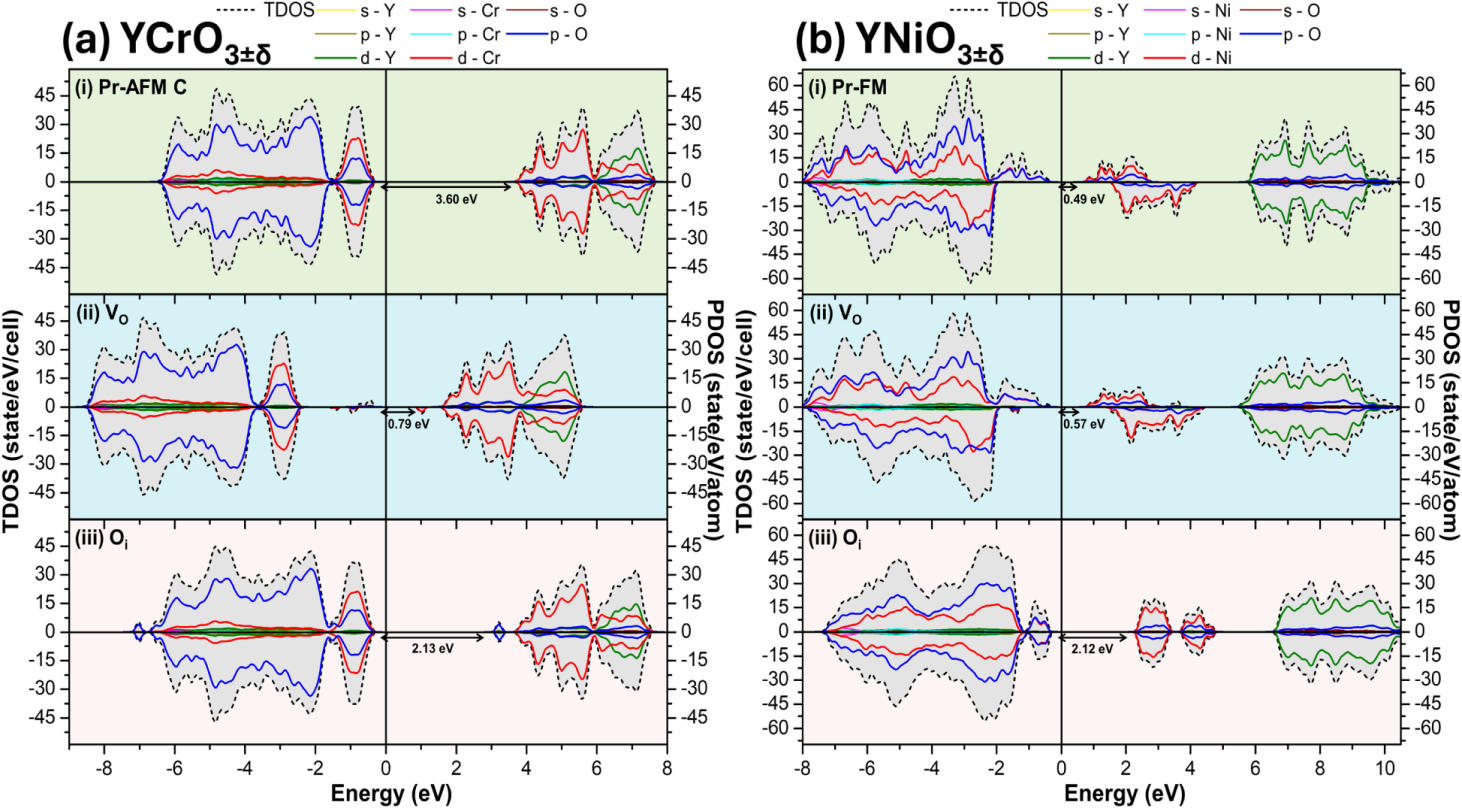
Calculated total (TDOS) and partial (PDOS) density of states for orthorhombic (a) YCrO_3±δ_ (i) Pr, (ii) V_O_ and (iii) O_i_ systems; and for (b) YNiO_3±δ_ (i) Pr, (ii) V_O_ and (iii) O_i_ systems. The results were obtained with the HSE06 hybrid functional and with the Fermi energy is set to zero.

The native defects, oxygen vacancy (V_O_) and interstitial oxygen (O_i_), were also investigated in the electronic structure of YCrO_3±δ_ perovskites for δ = 0.08 (2.08%). For this purpose, one oxygen atom was removed or added to a unit cell containing 80 oxygen atoms. The V_O_ defect was introduced at the 8d Wyckoff position, as tests performed for the inequivalent vacancy sites revealed that both sites exhibit equivalent formation energies and densities of states (Figure S5). Supercell calculations with 160 atoms for YTiO_3±δ_ indicated that an 80-atom supercell is sufficient to capture the local electronic effects of these isolated defects. The overall electronic configuration remains largely unchanged in the presence of V_O_ and O_i_, as shown in Figure 4a-ii and a-iii. However, new defect levels emerge within the bandgap, reducing its value. In the V_O_ structure, these midgap states are primarily associated with Cr *d*-orbitals. In the O_i_ structure, the conduction band minimum is mainly composed of the *p*-orbitals of the interstitial oxygen atom, suggesting the absence of a Cr–O_i_ bond.

The calculated electronic band gap for pristine YCrO_3_ using the HSE06 functional is 3.60 eV, which shows excellent agreement with the experimental value of 3.72 eV^60^. However, the introduction of native defects significantly reduces this value. For YCrO_3±δ_ containing V_O_, the band gap decreases to 0.79 eV, while for a system with O_i_ it is 2.13 eV. Notably, both defective structures preserve the AFM ordering of pristine material. This trend of band gap reduction in the presence of defects was observed for all YBO_3±δ_ materials studied, except for B = Ni. This trend is summarized in Figure 5, which plots the calculated band gaps for all YBO_3±δ_ systems as a function of the B-site cation. As illustrated, the presence of either V_O_ or O_i_ consistently leads to a reduction in the band gap compared to the pristine reference across the series, with the reduction being more pronounced in the V_O_ defect. This behavior is due to the introduction of defect levels in the band gap region. The calculated and reference electronic band gaps and total magnetic moment for all YBO_3±δ_ compounds, along with the influence of native point defects, are summarized in Table S4. The TDOS and PDOS figures are present in Figure S6 in Supporting Information section.

**Figure 5:**
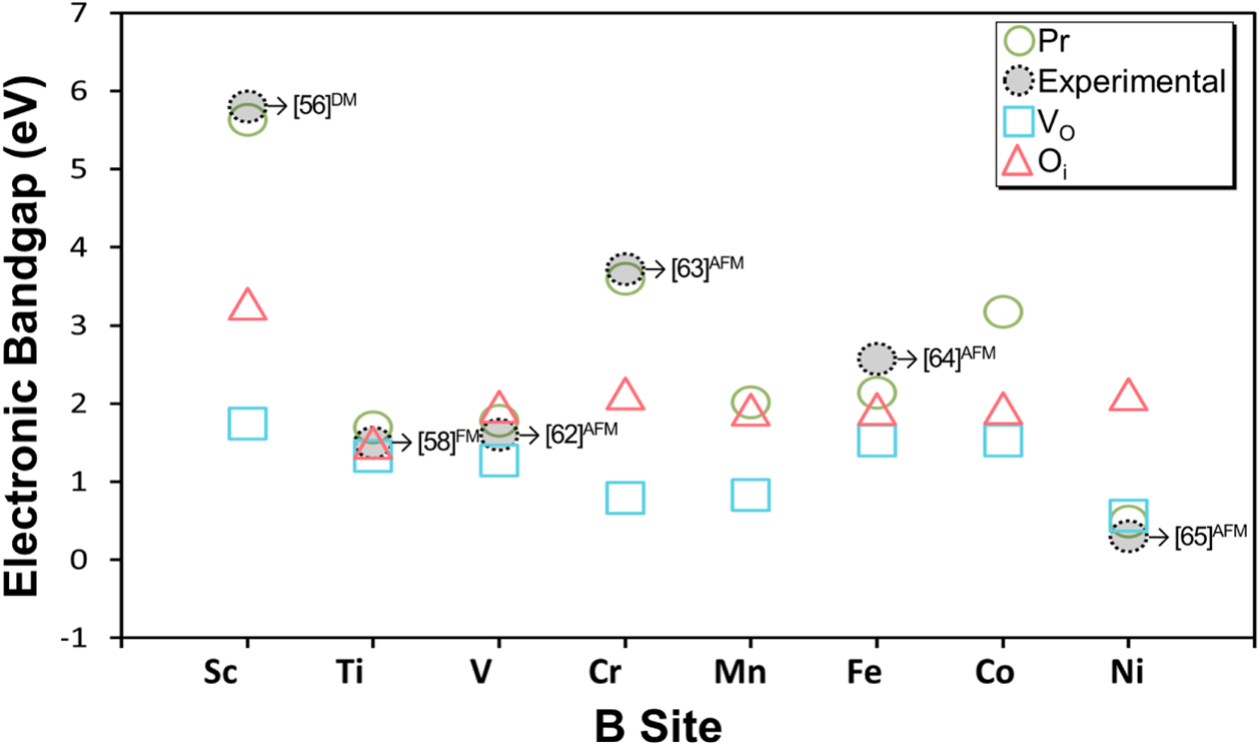
Calculated and experimental electronic band gap values (in eV) for YBO_3±δ_ perovskites (B = Sc, Ti, V, Cr, Mn, Fe, Co, Ni). The green circles correspond to DFT-calculated gaps for pristine structures under ferromagnetic (Pr-FM) and diamagnetic or antiferromagnetic (Pr-AFM/DM) configurations. The experimental values obtained from literature are shown with reference numbers indicated next to each data point.^56,60,62–65^

The magnitude of this reduction varies depending on the B-site cation and the type of defect. For instance, in YScO_3±δ_, the pristine band gap of approximately 5.63 eV is significantly reduced by both the O_i_ defect (∼3.28 eV) and the V_O_ defect (∼1.3 eV). The results also confirm that for nearly all materials, native defects can transition the material from a wide-gap semiconductor to a narrow-gap one. The main exception to this trend is YNiO_3±δ_ system. As illustrated in Figure 4 b-ii and b-iii, the electronic structure of YNiO_3_ exhibits a valence band primarily composed of O 2p states strongly hybridized with Ni 3d orbitals, while the conduction band minimum (CBM) is dominated by Ni 3d states. This orbital distribution reflects the strong Ni–O hybridization characteristic of negative charge transfer insulators.^61^ Upon the introduction of native oxygen defects, the overall band topology remains qualitatively similar, with O_i_ showing an AFM behavior; however, localized states associated with the modified Ni–O environment alter the bandgap magnitude. In particular, the V_O_ slightly increases the bandgap to 0.57 eV, whereas the O_i_ leads to a more significant widening (2.12 eV).

### Thermal Expansion Coefficient

The thermal properties of cathode materials are a critical factor for the stability and lifespan of SOFCs, given their high operating temperatures, which are often between 800 and 1200 K. In this context, ensuring the thermal stability of the material and achieving a compatible Thermal Expansion Coefficient (TEC) are fundamental. It is essential that the cathode material remains structurally cohesive and that its TEC matches that of the other cell components, such as the electrolyte, to prevent mechanical stress that can lead to fractures or device delamination.^12,14,66–68^

The TEC is one of the most important parameters for ensuring this compatibility. The TEC values, for the YBO_3±δ_ series, were obtained from isotropic MD simulations. In this method, the average volume of each structure was calculated, in isotropic simulations, over a temperature range of 600 to 1300 K. The TEC was then determined from the derivative of volume with respect to temperature (dV/dT), obtained via a linear regression of the simulated volume data.^69^ We can see that the O_i_ and Pr structures exhibit practically the same volume over the entire temperature range, whereas the structures containing V_O_ consistently show volumes approximately 0.5% higher. All volume versus temperature plots, together with their fitted curves, are presented in Figure S7 of the Supporting Information.

Figure 6 shows a strong dependence of the TEC on the B-site element. Notably, the YCrO_3±δ_ and YTiO_3±δ_ perovskites exhibit the highest TEC values, varying between 9.92 and 11.48 × 10^–6^ K^–1^ respectively. These results are promising as they are within the ideal range for major SOFC electrolytes, such as YSZ, which is 10.0 to 12.5 × 10^–6^ K^–1 12,14,19,66,68^. This thermomechanical compatibility suggests that YCrO_3±δ_ and YTiO_3 ± δ_ are good candidates for cathodes, capable of minimizing structural instability issues at high temperatures. In contrast, the other analyzed compounds, such as those with V, Co, and Ni at the B site, showed considerably lower TEC values in the range of 5.0 to 6.0 × 10^–6^ K^–1^, but still in the same order of magnitude.

**Figure 6:**
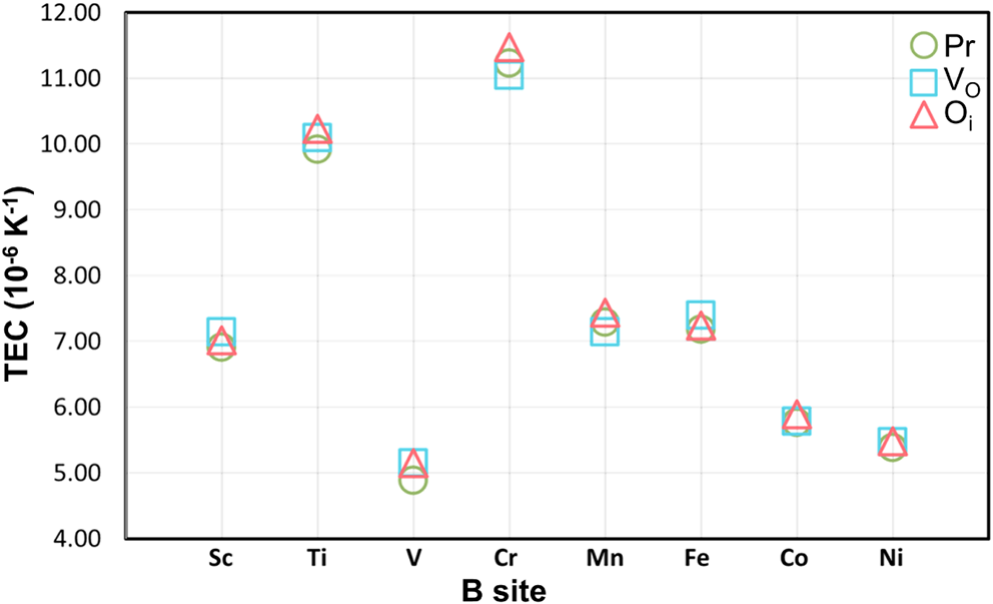
Linear thermal expansion coefficient (TEC) for each B-site in YBO_3±δ_ perovskites in Pr, V_O_ and O_i_ configurations, based on MD simulations. The values obtained in this work are comparable to the CTE values of major electrolytes used in SOFCs, which range from 10.0 to 12.5 × 10^–6^ K^–1^.^16,17^

Another important observation is that the presence of native defects, whether V_O_ or O_i_, at the studied concentration of 0.52%, does not significantly alter the TEC value. This is evidenced in the figure by the close clustering of the data points (Pr, V_O_ and O_i_) for each site element. Therefore, the dominant factor governing thermal expansion in these structures is the choice of the B site cation, rather than the presence of point defects at low concentrations.

### Oxygen Ionic Self-Diffusion

Ionic self-diffusion is a key process for the operation and efficiency of SOFCs. The cathode is often the main limiting factor in reducing the operating temperature of these devices, as the self-diffusion of O^2–^ ions through the crystal structure typically requires high temperatures. Therefore, discovering materials that exhibit high oxygen self-diffusion coefficients at lower temperatures (below 1100 K) is a primary goal for advancing SOFCs technology.^9,12,19,70,71^ The presence of native defects in the material structure, such as V_O_ and O_i_, is one of the main mechanisms that enable and promote this ionic self-diffusion.^16^

To investigate the motion of ions in MD simulations, the primary tool used is the calculation of the Mean Squared Displacement (MSD), in temperatures between 600 and 2000 K. The MSD measures the average of the squared distance that a set of particles travels over time from their initial positions. It is described by eq 3 The analysis of a MSD versus time plot identifies particle behavior. An MSD value that increases linearly with time indicates diffusive behavior, whereas a value that reaches a plateau indicates that the particles are only vibrating around their equilibrium positions without diffusing through the lattice.

The analysis revealed that the cations (Y^3+^ and B^3+^) are nondiffusive under any simulated conditions, with the results at 1000 K presented in Figure S8 as a representative case. Figure 7 presents the corresponding MSD results, in a logarithm scale, only for oxygen ions for the YScO_3±δ_ structure as an example, but all perovskites analyzed showed the same behavior (see Figure S9). In the Pr system showed in Figure 7a, the oxygen ions are also localized, showing no self-diffusion even at elevated temperatures. In stark contrast, the introduction of native defects enables significant oxygen ion self-diffusion. This is shown for systems containing V_O_ and O_i_ in Figure 7b and c respectively, where the MSD curves increase steadily over time. For both defective configurations, the rate of self-diffusion is strongly dependent on temperature, as expected, increasing substantially as thermal energy rises. A direct comparison of the defective systems in YScO_3±δ_ shows that oxygen self-diffusion is substantially more pronounced in the presence of O_i_ than with V_O_ at any given temperature.

**Figure 7:**
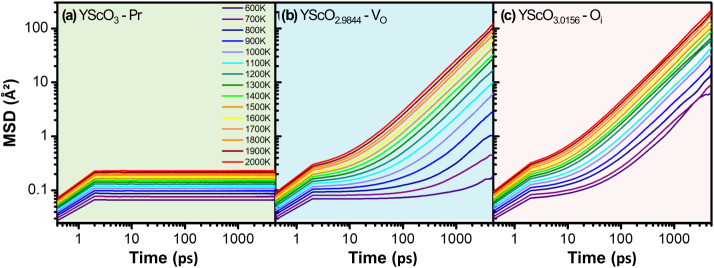
Mean squared displacement of oxygen ions for orthorhombic YScO_3±δ_ in (a) Pristine (Pr), (b) oxygen vacancy (V_O_), and (c) interstitial oxygen (O_i_) configurations, at temperatures ranging from 600 to 2000 K, based on MD simulations.

To further analyze the ionic transport, the self-diffusion coefficient (D_c_) and the activation energy (*E*_a_) for oxygen self-diffusion were calculated for each system. D_c_ was determined via linear regression with the Einstein relation (eq 4) in MSD along time results for each temperature. *E*_a_ represents the energy barrier that must be overcome to initiate the self-diffusion mechanism, making it a key parameter for characterizing ionic transport in the lattice. This value was determined by analyzing the temperature dependence of the D_c_ using the Arrhenius eq (eq 5).

Figure 8 shows the Arrhenius plot for YScO_3±δ_ as a representative example of the analysis that was performed for all YBO_3±δ_ perovskites. The plot clearly shows the linear behavior predicted by the Arrhenius equation for systems containing both V_O_ and O_i_, for this analysis, only data points above 800 K were used for the fitting, as self-diffusion is more effective in this range. From this analysis, the activation energy for YScO_3±δ_ with V_O_ was determined to be 0.52 eV, while for the system with O_i_, it was significantly lower at 0.32 eV. This trend was found to be consistent across the entire series of materials studied. This suggests a more energetically favorable pathway for oxygen ion migration in these YBO_3±δ_ perovskite structures containing O_i_ defects, consistent with previous detailed analyses.^16^

**Figure 8:**
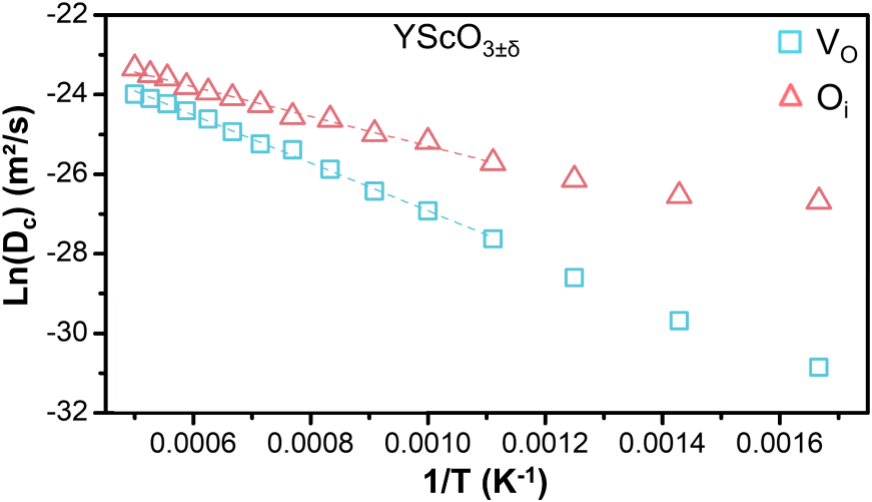
Arrhenius plots showing the logarithm of the self-diffusion coefficient (D_c_) versus inverse temperature (1/T) for orthorhombic YBO_3±δ_ containing oxygen vacancies (V_O_) and interstitial oxygen (O_i_), for different B-site cations, based on MD simulations. The fitting was performed using the equation $D_{c}=D_{0}\exp\left(\frac{-E_{a}}{k_{B}T}\right)$ , disregarding temperatures below 900 K.

To assess the influence of the B site cation on ionic transport in YBO_3±δ_ compounds, both the self-diffusion coefficient (D_c_) at 1000 K and the activation energy (*E*_a_) are presented in Figure 9a and b, respectively. As expected, according to eq 5, materials exhibiting lower activation energies tend to display higher self-diffusion coefficients. Among the compounds studied, YTiO_3±δ_ and YScO_3±δ_ stand out, showing the lowest *E*_a_ values and the highest D_c_, mainly for the interstitial oxygen case. Additionally, for almost every B site element, the O_i_-mediated mechanism consistently shows lower activation energies and higher self-diffusion coefficients compared to the V_O_ configuration. While comparisons between native defect types within the same force-field parameter set are robust, comparisons across different B-site cations should be regarded as a screening tool, since the results may be sensitive to the force-field fitting and require further validation using first-principles methods.

**Figure 9:**
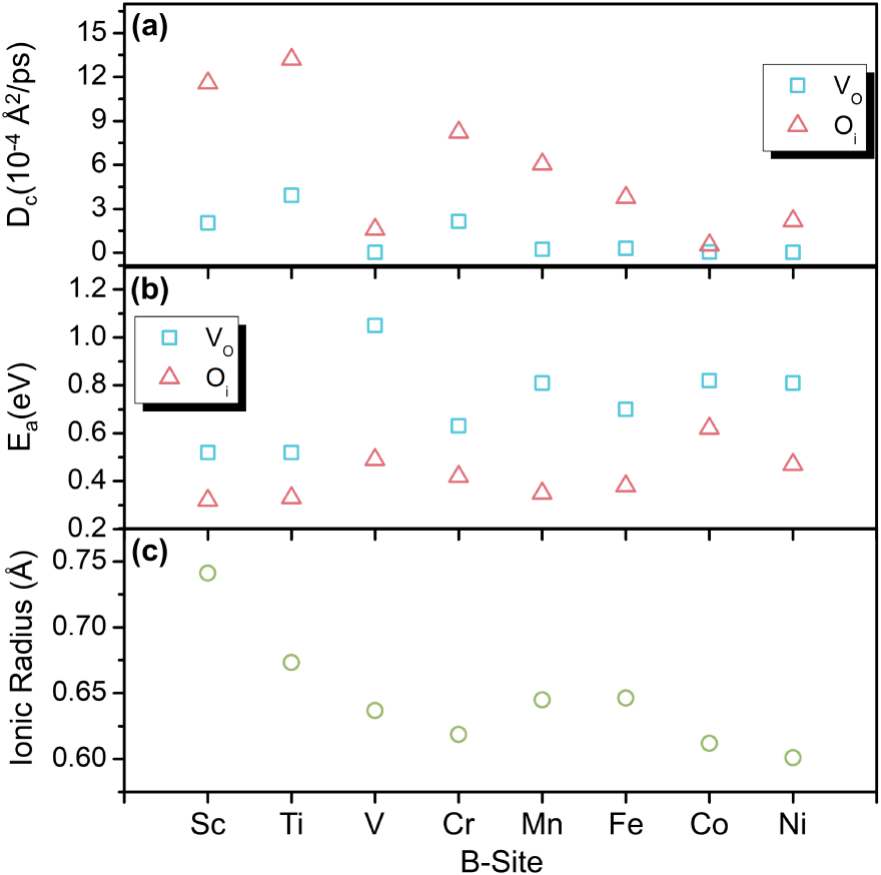
Dependence of (a) D_c_, at a temperature of 1000 K, (b) *E*_a_ from MD calculations for different B sites and (c) ionic radius of different B^3+^ ion. The ionic radius of dopants in YBO_3±δ_ perovskites, obtained from Alsalman et al.^72^

The mechanism underlying the superior oxygen self-diffusion via O_i_, as compared to oxygen vacancies V_O_, has been previously elucidated for YTiO_3±δ_.^16^ In this system, O_i_ self-diffusion occurs through an anisotropic migration pathway predominantly along the *y* direction, facilitated by distortions of the TiO_6_ octahedra. The process involves a correlated motion between the interstitial and nearby lattice oxygen atoms, forming a transient exchange configuration that enables efficient migration.

When extending this analysis to the broader YBO_3±δ_ series, it is important to note that such anisotropic behavior is not universal, although all the compounds, except for those with B = V and B = Ni, with O_i_ exhibit preferential diffusion along the y direction, it is necessary to perform a complementary analysis to better understand the associated mechanism in another systems containing both V_O_ and O_i_ defects, as the diffusion mechanism depends strongly on the specific B-site cation and its associated electronic and structural characteristics (see Figure S10). Differences in ionic radius among the B-site cations (Figure 9c) influence the lattice free volume available for O_i_ migration. Larger ionic radii can increase the free volume within the structure, which may facilitate the formation of favorable migration pathways for O_i_. Additionally, structural analyses show that these compounds exhibit varying degrees of B–O–B bond angle distortion, indicative of different octahedral tilting patterns (see Figure 3). While such distortions are necessary to enable O_i_ migration, they are not solely sufficient, since higher oxygen mobility also depends on the local free volume. In contrast, the V_O_-mediated self-diffusion mechanism involves random hopping of lattice O^2–^ ions into neighboring vacant sites and appears less sensitive to octahedral tilting [16] or ionic radius.

## Conclusions

In this study, we systematically investigated the structural, electronic, thermal, and ionic transport properties of orthorhombic YBO_3±δ_ perovskites (B = Sc, Ti, V, Cr, Mn, Fe, Co, Ni) using a combined computational approach of DFT and MD. Our findings highlight the importance of native defects, V_O_ and O_i_ in modulating the performance of these materials for SOFCs cathode applications.

The orthorhombic phase was found to be energetically more favorable than the cubic structure for all B-site cations. Its thermomechanical stability was confirmed through TEC calculations, particularly for YCrO_3±δ_ and YTiO_3±δ_, which exhibited coefficients closely aligned with the optimal range for YSZ-based electrolytes.

The electronic structure analysis using hybrid HSE06 functional revealed that pristine YBO_3_ perovskites behave as moderate-to-wide bandgap semiconductors. The introduction of V_O_ and O_i_ generally reduced the bandgap by creating defect states, however, in certain systems like YNiO_3‑δ_ and YMnO_3±δ_, an increase in the bandgap was observed.

Ionic self-diffusion simulations demonstrated that pristine structures show negligible oxygen mobility, while defect-containing systems exhibit substantial ion transport. O_i_ native defects consistently led to higher self-diffusion coefficients and lower activation energies compared to V_O_ in all compositions. Notably, YTiO_3±δ_ and YScO_3±δ_ displayed superior ionic conductivity in comparison to the other analyzed perovskites.

These results highlight the critical role of defect engineering in enhancing the functional performance of YBO_3±δ_ perovskites and contribute to a deeper understanding of the effects of native V_O_ and O_i_ defects at different B-site compositions. Furthermore, highlight the effectiveness of employing combined MD and DFT methodologies for the predictive design and analysis of advanced cathode materials.

## Supplementary Material



## Notes

General results for YBO_3_ with O_i_ and V_O_ defects from MD and DFT simulations; structural representative models; PAW pseudopotentials used; volume optimization curves; structural and electronic results; total and partial density of states; volume versus temperature curves; mean square displacement (MSD) total and directional analyses (PDF)

## References

[1] Höök M., Tang X. (2013). Depletion of Fossil Fuels and Anthropogenic Climate Change-A Review. Energy Policy.

[2] Achakulwisut P., Erickson P., Guivarch C., Schaeffer R., Brutschin E., Pye S. (2023). Global Fossil Fuel Reduction Pathways
under Different Climate Mitigation Strategies and Ambitions. Nat. Commun..

[3] Steele, B. C. H. ; Heinzel, A. Materials for Fuel-Cell Technologies, In Materials for Sustainable Energy; Macmillan Publishers Ltd: UK, 2010; Vol. 414, pp. 224–231. DOI: 10.1142/9789814317665_0031.

[4] Gohar O., Khan M. Z., Saleem M., Chun O., Babar Z. U. D., Rehman M. M. U., Hussain A., Zheng K., Koh J. H., Ghaffar A., Hussain I., Filonova E., Medvedev D., Motola M., Hanif M. B. (2024). Navigating the Future
of Solid Oxide
Fuel Cell: Comprehensive Insights into Fuel Electrode Related Degradation
Mechanisms and Mitigation Strategies. Adv. Colloid
Interface Sci..

[5] Ighodaro O. O., Scott K., Xing L. (2017). An Isothermal Study of the Electrochemical
Performance of Intermediate Temperature Solid Oxide Fuel Cells. J. Power Energy Eng..

[6] Jacobs R., Mayeshiba T., Booske J., Morgan D. (2018). Material Discovery
and Design Principles for Stable, High Activity Perovskite Cathodes
for Solid Oxide Fuel Cells. Adv. Energy Mater..

[7] Zhang J., Ricote S., Hendriksen P. V., Chen Y. (2022). Advanced Materials
for Thin-Film Solid Oxide Fuel Cells: Recent Progress and Challenges
in Boosting the Device Performance at Low Temperatures. Adv. Funct. Mater..

[8] Sun C., Hui R., Roller J. (2010). Cathode Materials for Solid Oxide Fuel Cells: A Review. J. Solid State Electrochem..

[9] Lyu Y., Xie J., Wang D., Wang J. (2020). Review of Cell Performance in Solid
Oxide Fuel Cells. J. Mater. Sci..

[10] Lee Y. L., Morgan D. (2012). Ab Initio and Empirical
Defect Modeling of LaMnO3±δ
for Solid Oxide Fuel Cell Cathodes. Phys. Chem.
Chem. Phys..

[11] Tarragó, D. P. ; Moreno, B. ; Chinarro, E. ; de Sousa, V. C. Perovskites Used In Fuel Cells Perovskite Materials - Synthesis, Characterisation, Properties, and Applications IntechOpen 2016 21 619 10.5772/61465

[12] Kilner J. A., Burriel M. (2014). Materials for Intermediate-Temperature Solid-Oxide
Fuel Cells. Annu. Rev. Mater. Res..

[13] Olsson E., Aparicio-Anglès X., De Leeuw N. H. (2016). Ab Initio Study
of Vacancy Formation in Cubic LaMnO3 and SmCoO3 as Cathode Materials
in Solid Oxide Fuel Cells. J. Chem. Phys..

[14] Olsson E., Aparicio-Anglès X., De Leeuw N. H. (2017). A Computational
Study of the Electronic Properties, Ionic Conduction, and Thermal
Expansion of Sm1-XAxCoO3 and Sm1-XAxCoO3-x/2 (A = Ba2+, Ca2+, Sr2+,
and x = 0.25, 0.5) as Intermediate Temperature SOFC Cathodes. Phys. Chem. Chem. Phys..

[15] Martins N. R., Borges D. D., Borges P. D. (2023). Computational Study of Native Defects
and Oxygen Diffusion in the YTiO3±δ as Cathode Materials
in SOFCs. J. Solid State Chem..

[16] Martins N. R., Scolfaro L., Damasceno
Borges P., Damasceno Borges D. (2025). Understanding
Oxygen Ion Diffusion Mechanisms in YTiO3 Structures with Native Defects. J. Phys. Chem. C.

[17] Tawse D. N., Fop S., Still J. W., Ballantyne O. J. B., Ritter C., Zhou Y., Dawson J. A., Mclaughlin A. C. (2025). Unlocking the Potential of Palmierite
Oxides: High Oxide Ion Conductivity via Induced Interstitial Defects. J. Am. Chem. Soc..

[18] Martins N. R., de Campos Viana L. A. F., Das Graças Santos A. A., Borges D. D., Welch E., Borges P. D., Scolfaro L. (2024). Computational
Study and Ion Diffusion Analyses of Native Defects and Indium Alloying
in β-Ga2O3 Structures. J. Vac. Sci. Technol.,
A.

[19] Chroneos A., Goulatis I. L., Solovjov A., Vovk R. V. (2024). The Evolution of
Solid Oxide Fuel Cell Materials. Appl. Sci..

[20] Savioli J., Watson G. W. (2020). Computational Modelling
of Solid Oxide Fuel Cells. Curr. Opin. Electrochem..

[21] Blöchl P. E. (1994). Projector
Augmented-Wave Method. Phys. Rev. B.

[22] Kresse G., Furthmüller J. (1996). Efficient
Iterative Schemes for Ab Initio Total-Energy
Calculations Using a Plane-Wave Basis Set. Phys.
Rev. B.

[23] Hohenberg P., Kohn W. (1964). Inhomogeneous Electron Gas. Phys. Rev..

[24] Kohn W., Sham L. J. (1965). Self-Consistent Equations Including Exchange and Correlation
Effects. Phys. Rev..

[25] Perdew J. P., Burke K., Ernzerhof M. (1996). Generalized
Gradient Approximation
Made Simple. Phys. Rev. Lett..

[26] Henderson T. M., Paier J., Scuseria G. E. (2011). Accurate
Treatment of Solids with
the HSE Screened Hybrid. Phys. Status Solidi
B Basic Res..

[27] Krukau A. V., Vydrov O. A., Izmaylov A. F., Scuseria G. E. (2006). Influence of the
Exchange Screening Parameter on the Performance of Screened Hybrid
Functionals. J. Chem. Phys..

[28] Boyd P. G., Moosavi S. M., Witman M., Smit B. (2017). Force-Field Prediction
of Materials Properties in Metal-Organic Frameworks. J. Phys. Chem. Lett..

[29] Thompson A. P., Aktulga H. M., Berger R., Bolintineanu D. S., Brown W. M., Crozier P. S., in’t Veld P. J., Kohlmeyer A., Moore S. G., Nguyen T. D., Shan R., Stevens M. J., Tranchida J., Trott C., Plimpton S. J. (2022). LAMMPS
- a Flexible Simulation Tool for Particle-Based Materials Modeling
at the Atomic, Meso, and Continuum Scales. Comput.
Phys. Commun..

[30] Grasselli F. (2022). Investigating
Finite-Size Effects in Molecular Dynamics Simulations of Ion Diffusion,
Heat Transport, and Thermal Motion in Superionic Materials. J. Chem. Phys..

[31] Kaub J., Kler J., Parker S. C., De Souza R. A. (2020). The Usefulness of
Molecular-Dynamics Simulations in Clarifying the Activation Enthalpy
of Oxygen-Vacancy Migration in the Perovskite Oxide BaTiO3. Phys. Chem. Chem. Phys..

[32] Veld P. J., Ismail A. E., Grest G. S. (2007). Application of Ewald Summations to
Long-Range Dispersion Forces. J. Chem. Phys..

[33] Khrapak S. A., Chaudhuri M., Morfill G. E. (2011). Freezing of Lennard-Jones-Type Fluids. J. Chem. Phys..

[34] Tosi M. P., Fumit F. G. (1964). Ionic Sizes and
Born Repulsive Parameters in the Nacl-Type
Alkali Halides-Ii the Generalized Huggins-Mayer Form*. J. Phys. Chem. Solids.

[35] Born M., Mayer J. E. (1932). Zur Gittertheorie Der Ionenkristalle. Zeitschrift Phys..

[36] Dutra J. D. L., Bispo T. D., de Freitas S. M., Rezende M. V. D. S. (2021). ParamGULP:
An Efficient Python Code for Obtaining Interatomic Potential Parameters
for General Utility Lattice Program. Comput.
Phys. Commun..

[37] Gale J. D., Rohl A. L. (2003). The General Utility Lattice Program (GULP). Mol. Simul..

[38] Gale J. D. (2005). GULP: Capabilities
and Prospects. Z. Kristallogr Cryst. Mater..

[39] Gale J. D. (1997). GULP: A
Computer Program for the Symmetry-Adapted Simulation of Solids. J. Chem. Soc., Faraday Trans..

[40] Gale J. D. (1996). Empirical
Potential Derivation for Ionic Materials. Philos.
Mag. B.

[41] Jain A., Ong S. P., Hautier G., Chen W., Richards W. D., Dacek S., Cholia S., Gunter D., Skinner D., Ceder G., Persson K. A. (2013). Commentary: The Materials Project:
A Materials Genome Approach to Accelerating Materials Innovation. APL Mater..

[42] Bitzek E., Koskinen P., Gähler F., Moseler M., Gumbsch P. (2006). Structural
Relaxation Made Simple. Phys. Rev. Lett..

[43] Clark J. B., Richter P. W., Toit L. D. (1978). High-Pressure
Synthesis of YScO3,
HoScO3, ErScO3, and TmScO3, and a Reevaluation of the Lattice Constants
of the Rare Earth Scandates. J. Solid State
Chem..

[44] Cao Y., Shafer P., Liu X., Meyers D., Kareev M., Middey S., Freeland J. W., Arenholz E., Chakhalian J. (2015). Magnetism
and Electronic Structure of YTiO3 Thin Films. Appl. Phys. Lett..

[45] Martínez-Lope M. J., Alonso J. A., Retuerto M., Fernéndez-Díaz M. T. (2008). Evolution
of the Crystal Structure of RVO3 (R = La, Ce, Pr, Nd, Tb, Ho, Er,
Tm, Yb, Lu, Y) Perovskites from Neutron Powder Diffraction Data. Inorg. Chem..

[46] Mall A. K., Paul B., Garg A., Gupta R. (2020). Temperature Dependent
X-Ray Diffraction and Raman Spectroscopy Studies of Polycrystalline
YCrO3 Ceramics across the TC ∼ 460 K. J. Raman Spectrosc..

[47] Iliev M. N., Abrashev M. V., Lee H.-G., Popov V. N., Sun Y. Y., Thomsen C., Meng R. L., Chu C. W. (1998). Raman Spectroscopy
of Orthorhombic Perovskitelike YMnO3 and LaMnO 3. Phys. Rev. B.

[48] Maiti R., Basu S., Chakravorty D. (2009). Synthesis
of Nanocrystalline YFeO3
and Its Magnetic Properties. J. Magn. Magn.
Mater..

[49] Buassi-Monroy O. S., Luhrs C. C., Chávez-Chávez A., Michel C. R. (2004). Synthesis of Crystalline YCoO3 Perovskite via Sol-Gel
Method. Mater. Lett..

[50] Alonso J. A., García-Muñoz J. L., Fernández-Díaz M. T., Aranda M. A. G., Martínez-Lope M. J., Casais M. T. (1999). Charge
Disproportionation in RNiO3 Perovskites: Simultaneous Metal-Insulator
and Structural Transition in YNiO3. Phys. Rev.
Lett..

[51] Arima T., Tokura Y. (1995). Optical Study of Electronic Structure in Perovskite-Type
RMO3 (R = La, Y; M = Sc, Ti, V, Cr, Mn, Fe, Co, Ni, Cu). J. Phys. Soc. Jpn..

[52] Sawada H., Morikawa Y., Terakura K., Hamada N. (1997). Jahn-Teller Distortion
and Magnetic Structures in LaMnO3. Phys. Rev.
B.

[53] Izyurov V. V., Nosov A. P., Gribov I. V., Andreeva M. A. (2023). Magnetic Phase Transitions
in Ultrathin YFeO3 Films According to Synchrotron Mössbauer
Reflectometry Data. Phys. Met. Metallogr..

[54] Chaturvedi V., Walter J., Paul A., Grutter A., Kirby B., Jeong J. S., Zhou H., Zhang Z., Yu B., Greven M., Mkhoyan K. A., Birol T., Leighton C. (2020). Strain-Induced
Majority Carrier Inversion in Ferromagnetic Epitaxial LaCoO3-δ
Thin Films. Phys. Rev. Mater..

[55] Marti X., Skumryev V., Cattoni A., Bertacco R., Laukhin V., Ferrater C., García-Cuenca M. V., Varela M., Sánchez F., Fontcuberta J. (2009). Ferromagnetism
in Epitaxial Orthorhombic
YMnO3 Thin Films. J. Magn. Magn. Mater..

[56] Yue J., Quackenbush N. F., Laraib I., Carfagno H., Hameed S., Prakash A., Thoutam L. R., Ablett J. M., Lee T. L., Greven M., Doty M. F., Janotti A., Jalan B. (2020). Electronic
Structure and Small-Hole Polarons in YTiO3. Phys. Rev. Mater..

[57] Garrett J. D., Greedan J. E., MacLean D. A. (1981). Crystal Growth and Magnetic Anisotropy
of YTiO3. Mater. Res. Bull..

[58] Mizokawa T., Fujimori A. (1996). Electronic Structure
and Orbital Ordering in Perovskite-Type
3 d Transition-Metal Oxides Studied by Hartree-Fock Band-Structure
Calculations. Phys. Rev. B.

[59] Varignon J., Bibes M., Zunger A. (2019). Origin of
Band Gaps in 3d Perovskite
Oxides. Nat. Commun..

[60] Jara A. N. L., Carvalho J. F., Júnior A. F., Maia L. J. Q., Santana R. C. (2018). On the
Optical and Magnetic Studies of YCrO3 Perovskites. Phys. B.

[61] Bisogni V., Catalano S., Green R. J., Gibert M., Scherwitzl R., Huang Y., Strocov V. N., Zubko P., Balandeh S., Triscone J. M., Sawatzky G., Schmitt T. (2016). Ground-State
Oxygen
Holes and the Metal-Insulator Transition in the Negative Charge-Transfer
Rare-Earth Nickelates. Nat. Commun..

[62] Lu C., Lee C. H., Nishimura T., Toriumi A. (2015). Yttrium Scandate Thin
Film as Alternative High-Permittivity Dielectric for Germanium Gate
Stack Formation. Appl. Phys. Lett..

[63] Tsvetkov A. A., Mena F. P., Ren Y., Elfimov I. S., van Loosdrecht P. H.
M., van der Marel D., Nugroho A. A., Menovsky A. A., Sawatzky G. A. (2002). Optical and Magneto-Optical
Study of Orbital and Spin
Ordering Transitions in YVO3. Phys. B.

[64] Zahrae
Kassimi F., Zaari H., Benyoussef A., El Kenz A. (2023). Electronic and Magneto-Electric Properties of YFeO3. J. Magn. Magn. Mater..

[65] Malyi O. I., Zunger A. (2023). Rise and Fall of Mott Insulating Gaps InYNiO3 Paramagnets
as a Reflection of Symmetry Breaking and Remaking. Phys. Rev. Mater..

[66] Vinchhi P., Khandla M., Chaudhary K., Pati R. (2023). Recent Advances on
Electrolyte Materials for SOFC: A Review. Inorg.
Chem. Commun..

[67] Shah N., Xu X., Love J., Wang H., Zhu Z., Ge L. (2024). Mitigating
Thermal Expansion Effects in Solid Oxide Fuel Cell Cathodes: A Critical
Review. J. Power Sources.

[68] Mahato N., Banerjee A., Gupta A., Omar S., Balani K. (2015). Progress in
Material Selection for Solid Oxide Fuel Cell Technology: A Review. Prog. Mater. Sci..

[69] Orlovskaya N., Lugovy M., Pathak S., Steinmetz D., Lloyd J., Fegely L., Radovic M., Payzant E. A., Lara-Curzio E., Allard L. F., Kuebler J. (2008). Thermal and Mechanical
Properties of LaCoO3 and La0.8Ca0.2CoO3 Perovskites. J. Power Sources.

[70] Sikstrom D., Thangadurai V. (2024). A Tutorial
Review on Solid Oxide Fuel Cells: Fundamentals,
Materials, and Applications. Ionics.

[71] Wu Z., Zhu P., Huang Y., Yao J., Yang F., Zhang Z., Ni M. (2025). A Comprehensive Review of Modeling
of Solid Oxide Fuel Cells: From
Large Systems to Fine Electrodes. Chem. Rev..

[72] Alsalman M., Alghofaili Y. A., Baloch A. A. B., Alsadah H., Alsaui A. A., Alqahtani S. M., Muqaibel A. H., Alharbi F. H. (2023). Outliers in Shannon’s
Effective Ionic Radii Table and the Table Extension by Machine Learning. Comput. Mater. Sci..

