# Supplementary material for: Influence of Native Defects on the Structural, Electronic, Thermal, and Ionic Transport Properties of YBO3 ± δ Perovskites
Source: Inorg Chem. 2026 Jan 23;65(5):2717–27. doi: 10.1021/acs.inorgchem.5c03650 (PMC12892329; doi:10.1021/acs.inorgchem.5c03650)
Supplement: Supplementary file 1 [file ic5c03650_si_001.pdf]

SUPORTING INFORMATION

Influence of Native Defects on the structural, electronic, thermal, and ionic transport properties of $YBO_{3\pm\delta}$ Perovskites

Nathan Rabelo Martins[1,2,3,*], Alan Antônio das Graças Santos[1], Luiz Augusto Ferreira de Campos Viana[3,4], Luisa Scolfaro[3], Daiane Damasceno Borges[2], Pablo Damasceno Borges[5],

AFFILIATIONS

[1] Instituto de Ciências Exatas e Tecnológicas, Universidade Federal de Viçosa, Campus Rio Paranaíba, Rodovia MG 230, km 7, Rio Paranaíba, MG, 38810-000, Brazil.

[2] Instituto de Física, Universidade Federal de Uberlândia, Av. João Naves de Ávila 2121, Campus Santa Mônica, Uberlândia, MG, 38400-902, Brazil.

[3] Department of Physics, Texas State University, San Marcos, TX, 78666, USA.

[4] Instituto Federal de Educação, Ciência e Tecnologia de Minas Gerais (IFMG) - Campus Avançado Arcos, Av. Juscelino Kubitschek 485, Arcos, MG, 35588-000, Brazil.

[5] Department of Materials Science, Military Institute of Engineering (IME), Praça General Tibúrcio 80, Urca, Rio de Janeiro, RJ, 22290-270, Brazil.

[*] electronic mail: nathanrabelo@ufv.br

Table S1. PAW pseudopotentials and corresponding valence states included in the VASP PBE dataset, used for the DFT calculations of YBO_3 perovskites.

Element	PAW Potential	Valence states included
**Y**	Y_sv	$4s^2\ 4p^6\ 4d^1\ 5s^2$
**Sc**	Sc_sv	$3s^2\ 3p^6\ 3d^1\ 4s^2$
**Ti**	Ti_sv	$3s^2\ 3p^6\ 3d^2\ 4s^2$
**V**	V_sv	$3s^2\ 3p^6\ 3d^3\ 4s^2$
**Cr**	Cr_sv	$3s^2\ 3p^6\ 3d^5\ 4s^1$
**Mn**	Mn_sv	$3s^2\ 3p^6\ 3d^5\ 4s^2$
**Fe**	Fe_sv	$3s^2\ 3p^6\ 3d^6\ 4s^2$
**Co**	Co_sv	$3s^2\ 3p^6\ 3d^7\ 4s^2$

**Ni**	Ni	$3d^8\ 4s^2$
**O**	O_s	$2s^2\ 2p^4$

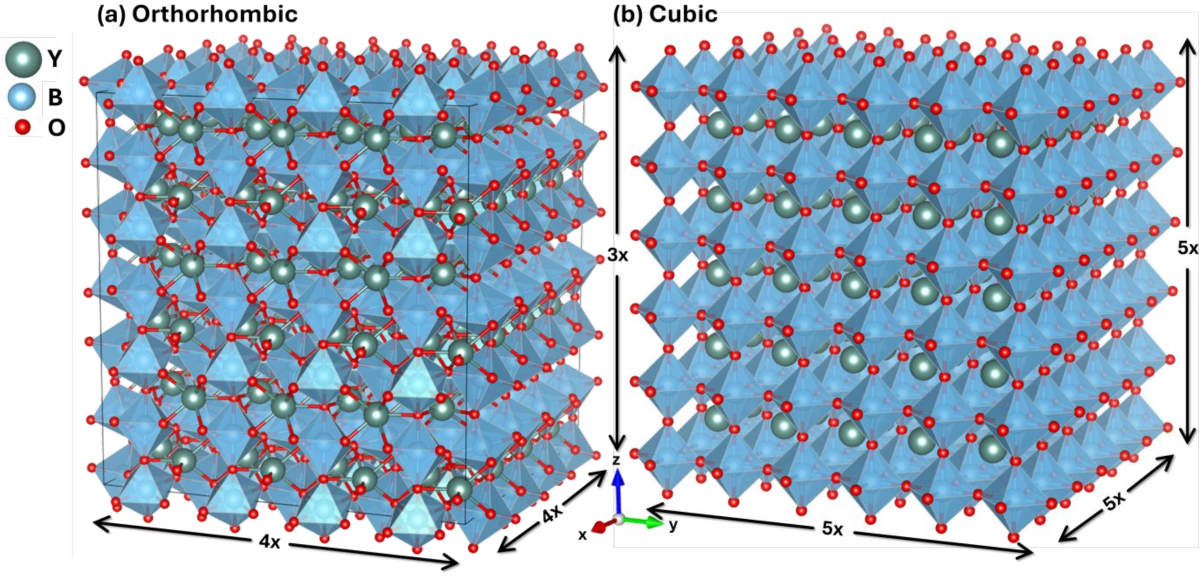

Figure S1. Representative MD (a) 4×4×3 supercell of the orthorhombic structure (960 atoms) and (b) 5×5×5 supercell of the cubic structure (625 atoms).

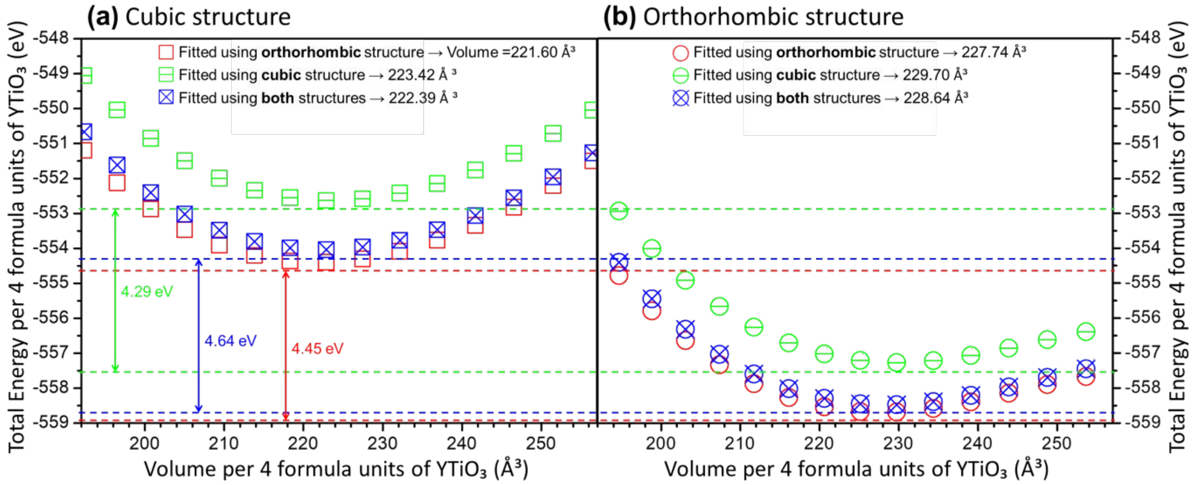

Figure S2. Total energy as a function of volume referring to a cell with 4 formula units of $YTiO_3$, using (a) the cubic and (b) the orthorhombic crystal structures. Each curve corresponds to a different set of Buckingham parameters fitted using distinct structural references: orthorhombic, cubic, or both. In all cases, the Ti_2O_3 oxide was included as part of the fitting procedure.

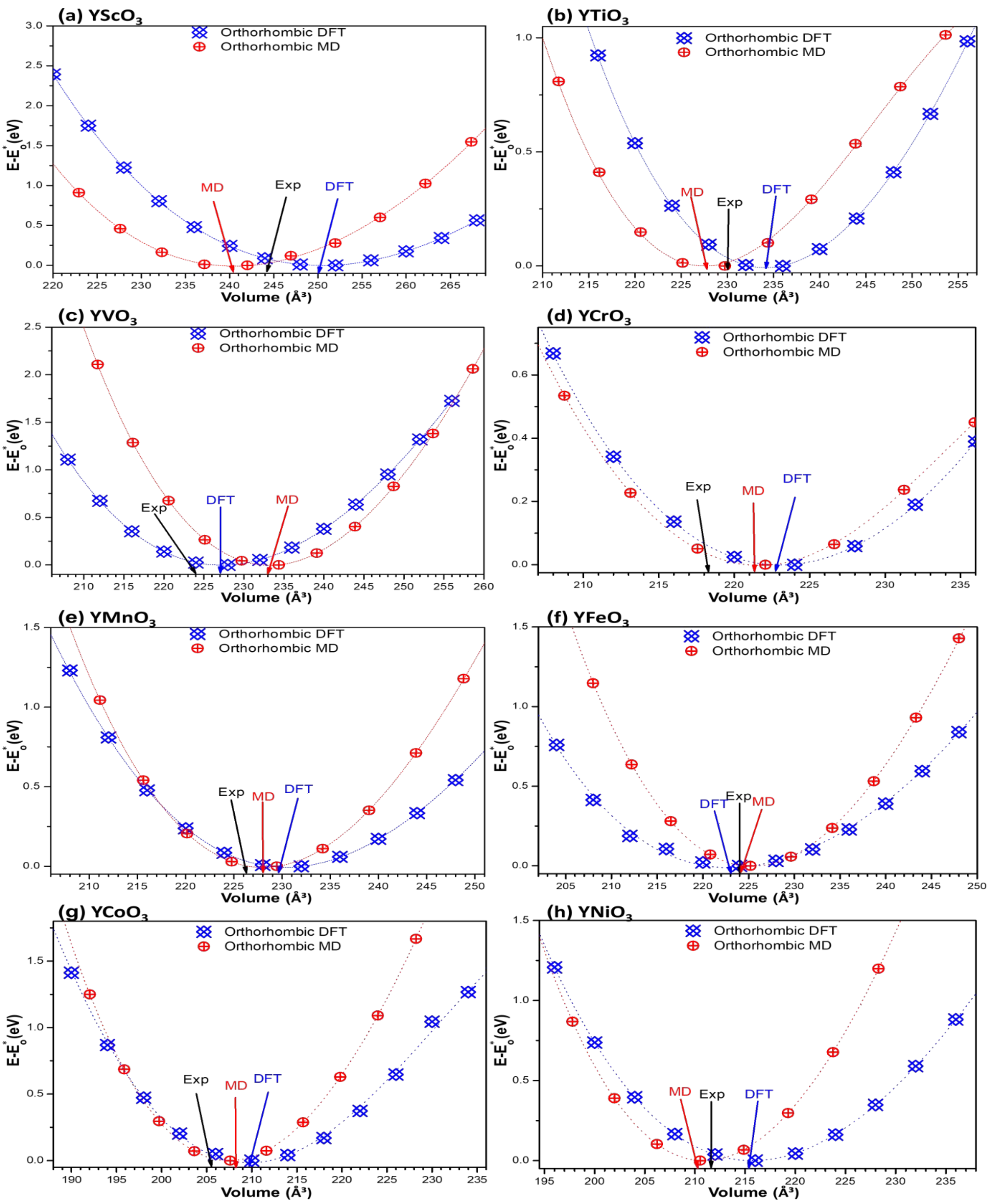

Figure S3. Total energy per unit cell with 20 atoms as a function of volume for the orthorhombic phase of YBO_3 compounds, considering different B-site cations: (a) Sc, (b) Ti, (c) V, (d) Cr, (e) Mn, (f) Fe, (g) Co, and (h) Ni. Results are obtained from DFT calculations (blue curves) and from classical structural relaxations (red curves). The minimum energy point for each method is highlighted to allow comparison of the predicted equilibrium volumes.

Table S2. Structural and elastic properties of YBO_3 compounds in the orthorhombic phase for B = Sc, Ti, V, Cr, Mn, Fe, Co, and Ni, obtained from DFT calculations, classical structural relaxations, and available experimental data. The listed parameters include the equilibrium volume, lattice constants a, b and c, the bulk modulus, and average interatomic distances for Y-O, B-O, and O-O pairs.

	Reference	Volume (Å^3)	a (Å)	b (Å)	c (Å)	*Bulk Modulus* (GPa)	Y-O (Å)	B-O (Å)	O-O (Å)
**$YScO_3$**	**DFT**	250.47	5.46	5.75	7.98	166	2.27	2.13	2.90
	**MD**	240.49	5.38	5.67	7.87	200	2.25	2.07	2.91
	**EXP** [1,2]	244.89	5.42	5.71	7.89	-	2.24	2.09	-
**$YTiO_3$**	**DFT**	234.2	5.36	5.66	7.72	181	2.32	2.05	2.91
	**MD**	227.86	5.36	5.61	7.65	207	2.25	2.01	2.85
	**EXP** [3–5]	229.77	5.32	5.68	7.61	163	2.22	2.02	-
**YVO_3**	**DFT**	227.26	5.32	5.63	7.6	189	2.25	2.03	2.86
	**MD**	233.18	5.35	5.65	7.7	290	2.25	2.07	2.91
	**EXP** [6,7]	224.02	5.28	5.59	7.58	-	-	2.02	-
**$YCrO_3$**	**DFT**	223.06	5.28	5.56	7.59	188	2.28	2.00	2.84
	**MD**	221.78	5.27	5.55	7.58	205	2.25	1.95	2.79
	**EXP** [8,9]	218.3	5.25	5.52	7.54	-	2.25	2.02	-
**$YMnO_3$**	**DFT**	229.54	5.28	5.85	7.42	169	2.25	2.06	2.86
	**MD**	228.07	5.27	5.84	7.41	235	2.25	2.01	2.85
	**EXP** [10,11]	226.47	5.26	5.84	7.36	-	-	1.93	-
**$YFeO_3$**	**DFT**	223.03	5.18	5.74	7.27	165	2.30	2.04	2.84
	**MD**	224.11	5.22	5.78	7.34	208	2.25	1.98	2.82
	**EXP** [12,13]	224.05	5.27	5.58	7.6	-	-	2.03	-
**$YCoO_3$**	**DFT**	209.9	5.13	5.68	7.21	184	2.37	1.95	2.79
	**MD**	208.33	5.13	5.68	7.21	285	2.30	1.95	2.67
	**EXP** [14,15]	204.8	5.13	5.42	7.37	-	-	1.95	-
**$YNiO_3$**	**DFT**	215.41	5.2	5.56	7.45	176	2.26	1.91	2.73
	**MD**	210.3	5.15	5.5	7.38	304	2.25	1.95	2.73
	**EXP** [16]	211.82	5.18	5.52	7.42	-	-	1.94	-

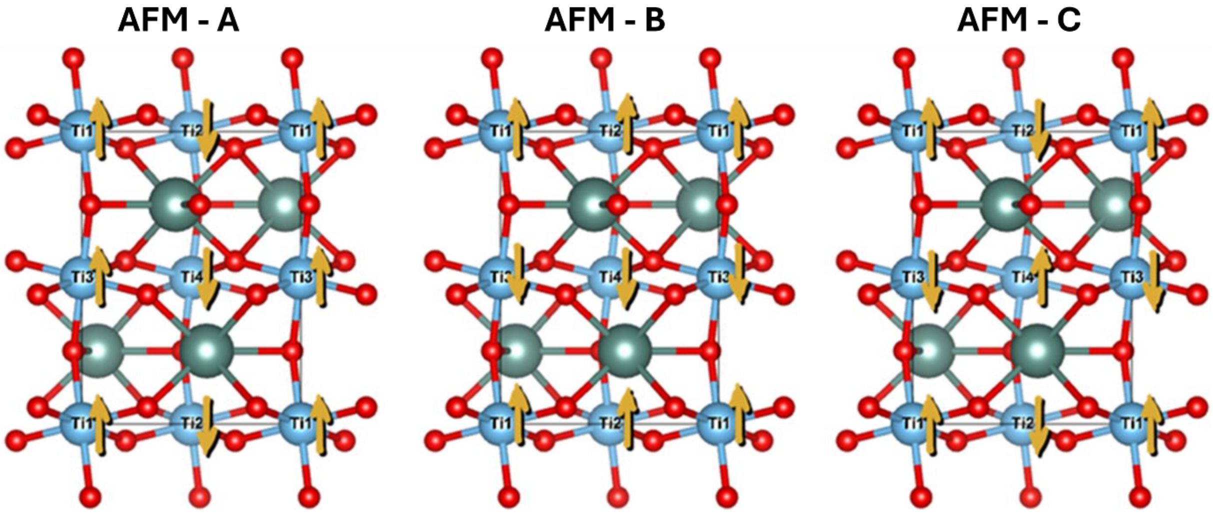

Figure S4. Three different antiferromagnetic configurations studied for the orthorhombic structures of YBO_3. The upward arrows represent spin up and the downward arrows represent spin down for each atom at site B, represented in the figure by the Ti atoms.

Table S3. Valence electronic configurations for neutral and with charge 3+, partial magnetic moments (μ_B) for B atoms, magnetic behavior (FM: ferromagnetic; AFM: antiferromagnetic; DM: diamagnetic) and energy difference between the magnetic configurations of orthorhombic YBO_3 compounds as a function of the B-site element. Magnetic moments were obtained from spin-polarized GGA-PBE DFT calculations.

B-site element	Neutral Valence Electron Configuration	B^{3+} Valence Electron Configuration	Partial magnetic moment (μ_B)	Magnetic ordering	ΔE (meV)
**Sc**	$4s^2\ 3d^1$	$3d^0$	**0**	DM	-
**Ti**	$4s^2\ 3d^2$	$3d^1$	**1**	FM	50
**V**	$4s^2\ 3d^3$	$3d^2$	**2**	FM	80
**Cr**	$4s^2\ 3d^4$	$3d^3$	**3**	AFM C	152
**Mn**	$4s^2\ 3d^5$	$3d^4$	**4**	AFM A	280
**Fe**	$4s^2\ 3d^6$	$3d^5$	**3**	AFM A	219
**Co**	$4s^2\ 3d^7$	$3d^6$	**0**	DM	-
**Ni**	$4s^2\ 3d^8$	$3d^7$	**1**	FM	51

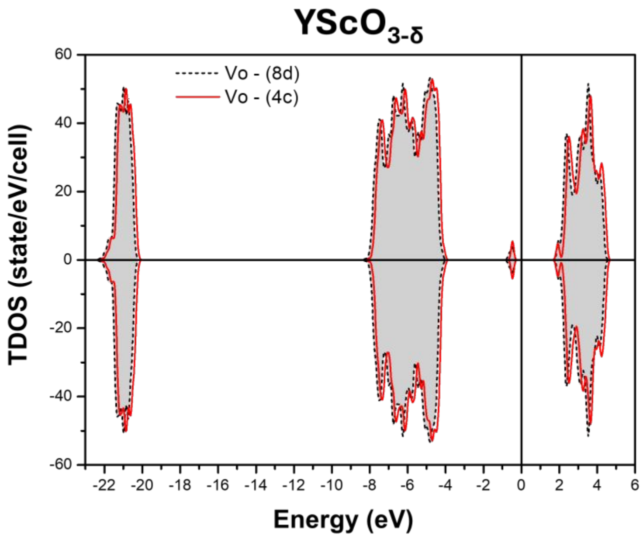

Figure S5. Calculated total density of states (TDOS) for orthorhombic (a) V_O-$YScO_{3-\delta}$ with 80 atoms, obtained with the HSE06 hybrid functional, for both inequivalent oxygen vacancy positions. The Fermi energy is set to zero.

Table S4. HSE06 calculated total magnetic moment and electronic band gap values (in eV) for orthorhombic YBO_3 (B = Sc, Ti, V, Cr, Mn, Fe, Co, Ni) perovskites. Pr refers to calculations for the pristine structure under the most favorable magnetic configuration. V_O and O_i represent the band gaps for structures containing oxygen vacancies and interstitial oxygen atoms, respectively. Bandgap Experimental values were extracted from the literature.

B-site element	Total Magnetic Moment	Bandgap HSE06	Bandgap Experimental Reference
**Sc-Pr**	0	5.63^{DM}	5.80 [17]^{DM}
**Sc-V_O**	0	1.74	
**Sc-O_i**	0	3.28	
**Ti-Pr**	16	1.70^{FM}	1.50 [18]^{FM}
**Ti-V_O**	16	1.33	
**Ti-O_i**	14	1.50	
**V-Pr**	32	1.78^{FM}	1.60 [19]^{AFM}
**V-V_O**	32	1.27	
**V-O_i**	22	1.96	
**Cr-Pr**	0	$3.60^{AFM\ C}$	3.72 [20]^{AFM}

**Cr-V_O**	0	0.79	
**Cr-O_i**	0	2.13	
**Mn-Pr**	0	2.02$^{AFM\ A}$	**-**
**Mn-V_O**	0	0.83	
**Mn-O_i**	8	1.92	
**Fe-Pr**	0	2.14$^{AFM\ A}$	2.57 [22]^{AFM}
**Fe-V_O**	2	1.53	
**Fe-O_i**	2	1.94	
**Co-Pr**	0	3.18^{DM}	-
**Co-V_O**	0	1.53	
**Co-O_i**	0	1.94	
**Ni-Pr**	16	0.25^{AFM}	0.3 [22]^{AFM}
**Ni-V_O**	16	0.57	
**Ni-O_i**	0	2.12	

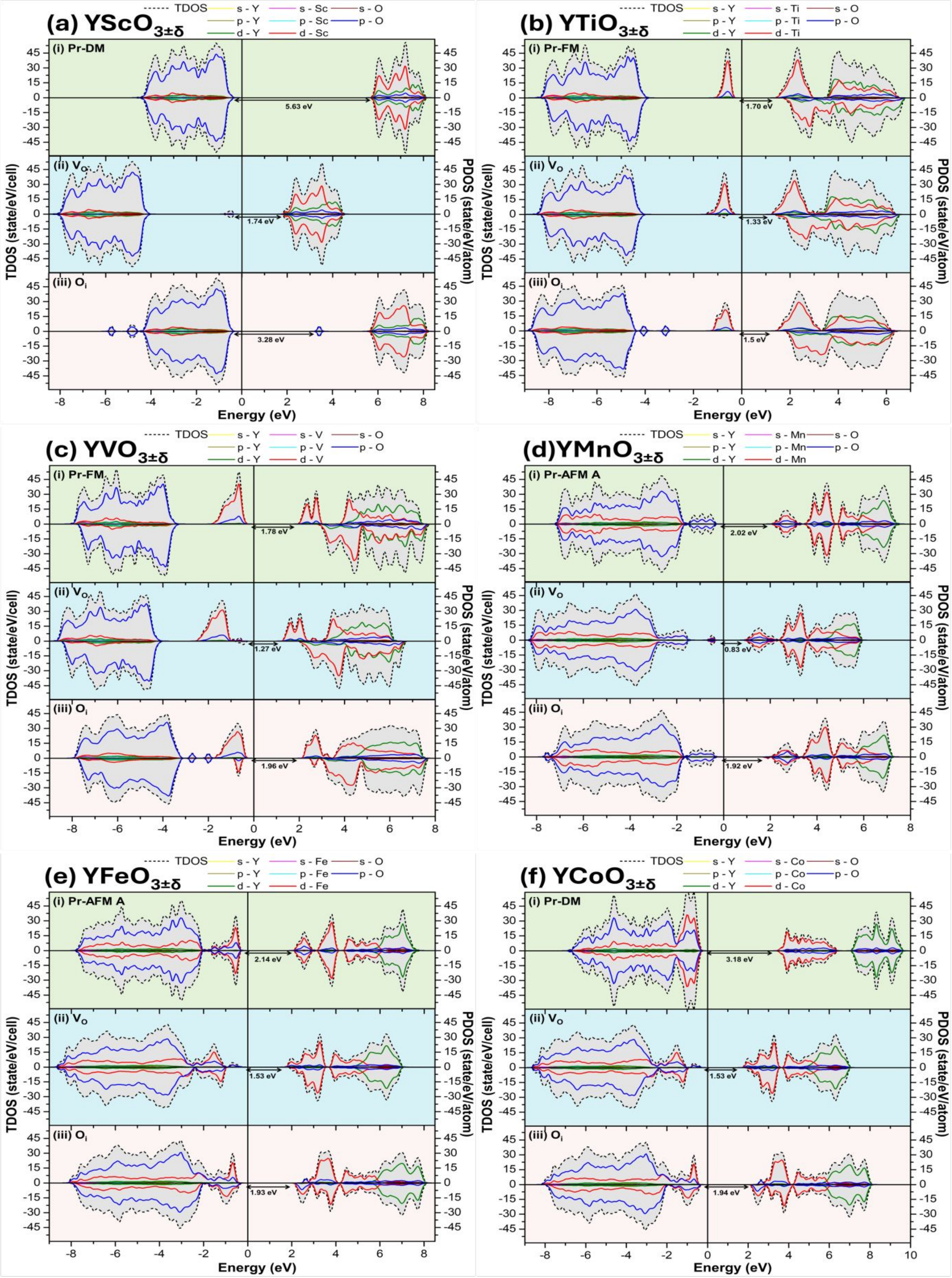

Figure S6. Calculated total (TDOS) and partial (PDOS) density of states obtained with the HSE06 hybrid functional in DFT calculations, for orthorhombic YBO_3 in (i) Pr and with systems containing native point defects (ii) V_O and (iii) O_i, for B = (a) Sc, (b) Ti, (c) V, (d) Mn, (e) Fe and (f) Co. The Fermi energy is set to zero.

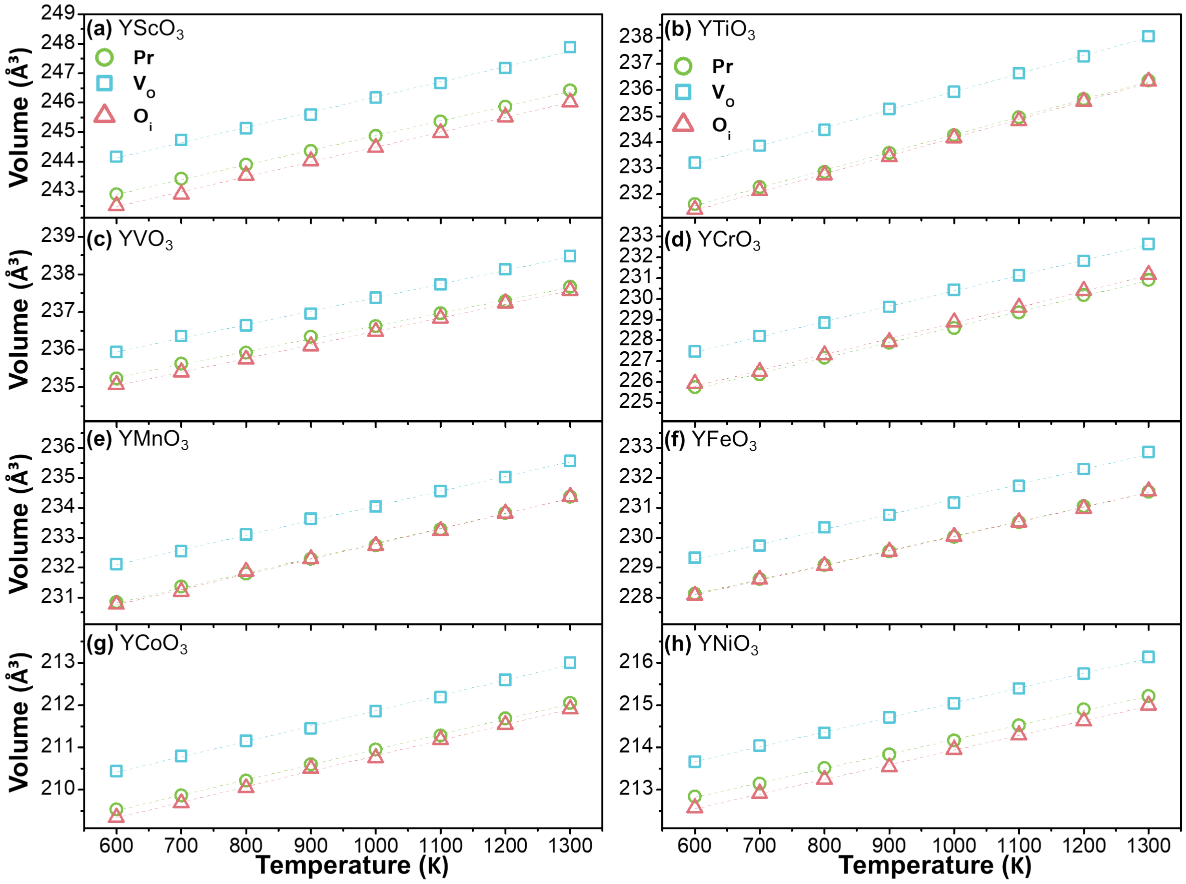

Figure S7. Calculated volume vs. temperature for the orthorhombic phase of YBO_3 compounds, considering different B-site cations: (a) Sc, (b) Ti, (c) V, (d) Cr, (e) Mn, (f) Fe, (g) Co, and (h) Ni under Pr, V_O, and O_i configurations in MD simulations between 600 and 1300K.

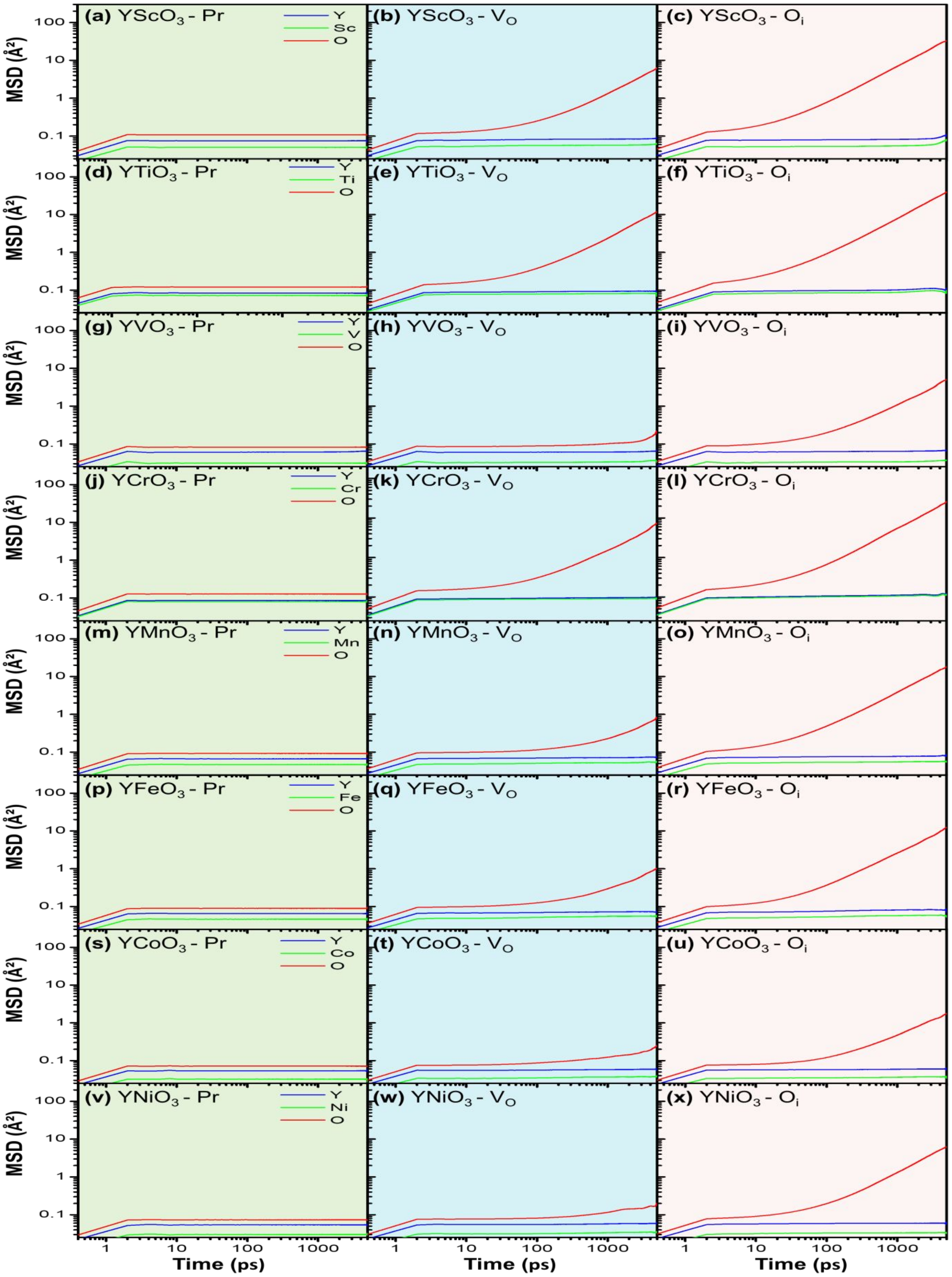

Figure S8. Mean square displacement (MSD) plots for ionic diffusion in $YBO_{3\pm\delta}$ compounds for Pr (green panels) and containing V_O (blue panels) and O_i, (red panels) at 1000K in MD simulations. The panels correspond to B = Sc (a)–(c), Ti (d)–(f), V (g)–(i), Cr (j)–(l), Mn (m)–(o), Fe (p)–(r), Co (s)–(u), and Ni (v)–(x).

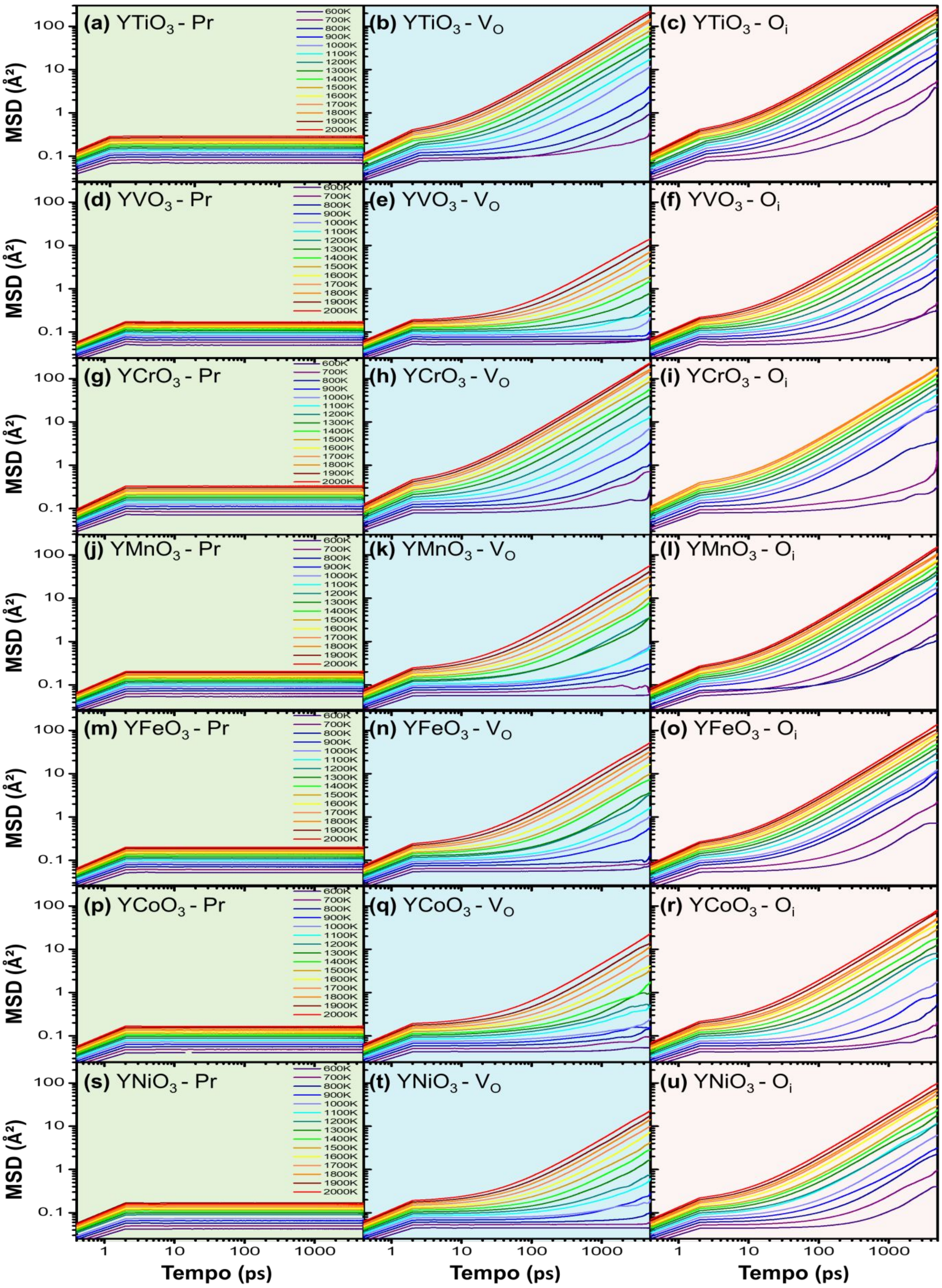

Figure S9. Mean squared displacement of oxygen ions for orthorhombic $YBO_{3\pm\delta}$ compounds for Pr (green panels) and containing V_O (blue panels) and O_i, (red panels), at temperatures ranging from 600 K to 2000 K, based on MD simulations.

The panels correspond to B = Sc (a)–(c), Ti (d)–(f), V (g)–(i), Cr (j)–(l), Mn (m)–(o), Fe (p)–(r), Co (s)–(u), and Ni (v)–(x).

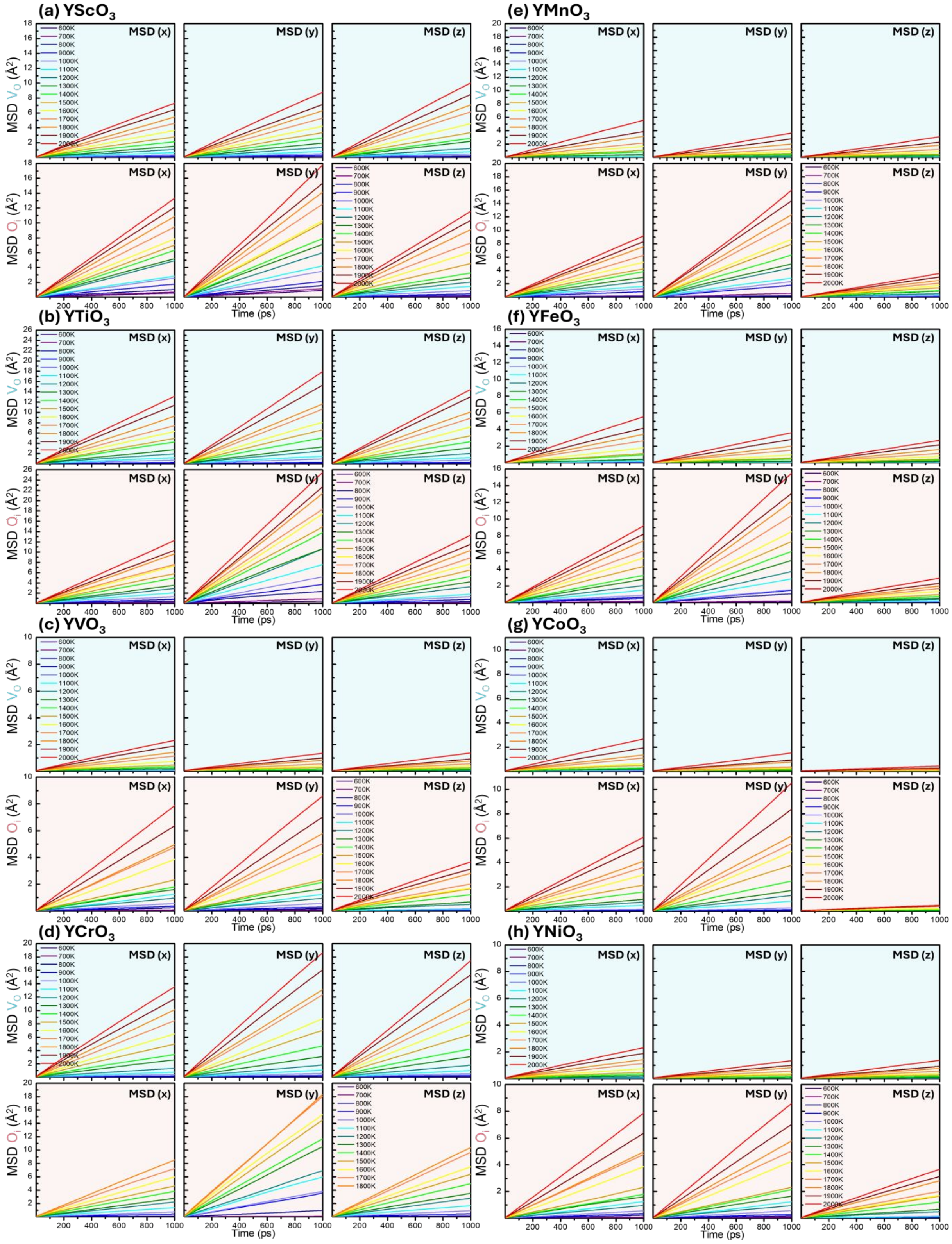

Figure S10. Directional mean square displacement (MSD) plots for oxygen diffusion in $YBO_{3\pm\delta}$ compounds containing V_O (blue panels) and O_i (red panels), at

temperatures ranging from 600 K to 2000 K. Each figure corresponds to a distinct B-site cation being B = (a) Sc, (b) Ti, (c) V, (d) Cr, (e) Mn, (f) Fe, (g) Co and (h) Ni, in MD simulations.

REFERENCES

[1] J.B. Clark, P.W. Richter, L.D. Toit, High-pressure synthesis of YScO3, HoScO3, ErScO3, and TmScO3, and a reevaluation of the lattice constants of the rare earth scandates, J Solid State Chem 23 (1978) 129–134. https://doi.org/10.1016/0022-4596(78)90058-0.

[2] S. Balamurugan, U.Ch. Rodewald, T. Harmening, L. van Wüllen, D. Mohr, H. Deters, H. Eckert, R. Pöttgen, PbO / PbF2 Flux Growth of YScO3 and LaScO3 Single Crystals – Structure and Solid-State NMR Spectroscopy, Zeitschrift Für Naturforschung B 65 (2010) 1199–1205. https://doi.org/10.1515/znb-2010-1004.

[3] Y. Cao, P. Shafer, X. Liu, D. Meyers, M. Kareev, S. Middey, J.W. Freeland, E. Arenholz, J. Chakhalian, Magnetism and electronic structure of YTiO3 thin films, Appl Phys Lett 107 (2015). https://doi.org/10.1063/1.4931039.

[4] I. Loa, X. Wang, K. Syassen, H. Roth, T. Lorenz, H. Hanfland, Y.-L. Mathis, Crystal structure and the Mott–Hubbard gap in YTiO3 at high pressure, Journal of Physics: Condensed Matter 23 (2007) 469601. https://doi.org/10.1088/0953-8984/23/46/469601.

[5] D.A. MacLean, H.-N. Ng, J.E. Greedan, Crystal structures and crystal chemistry of the RETiO3 perovskites: RE = La, Nd, Sm, Gd, Y, J Solid State Chem 30 (1979) 35–44. https://doi.org/10.1016/0022-4596(79)90127-0.

[6] M.J. Martínez-Lope, J.A. Alonso, M. Retuerto, M.T. Fernéndez-Díaz, Evolution of the crystal structure of RVO3 (R = La, Ce, Pr, Nd, Tb, Ho, Er, Tm, Yb, Lu, Y) perovskites from neutron powder diffraction data, Inorg Chem 47 (2008) 2634–2640. https://doi.org/10.1021/ic701969q.

[7] M. Reehuis, C. Ulrich, P. Pattison, B. Ouladdiaf, M.C. Rheinstädter, M. Ohl, L.P. Regnault, M. Miyasaka, Y. Tokura, B. Keimer, Neutron diffraction study of YVO3, NdVO3, and TbVO3, Phys Rev B Condens Matter Mater Phys 73 (2006). https://doi.org/10.1103/PhysRevB.73.094440.

[8] S. Geller, Crystallographic studies of perovskite-like compounds. IV. Rare earth scandates, vanadites, galliates, orthochromites, Acta Crystallogr 10 (1957) 243–248. https://doi.org/10.1107/s0365110x57000778.

[9] S. Yue, Y. Jing, Y. Sun, R. Huang, Z. Wang, J. Zhao, N.R. Aluru, Multi-scale simulation of anisotropic fracture behavior in BaZrO3, Appl Phys A Mater Sci Process 128 (2022). https://doi.org/10.1007/s00339-022-06023-9.

[10] M.N. Iliev, M. V Abrashev, H.-G. Lee, V.N. Popov, Y.Y. Sun, C. Thomsen, R.L. Meng, C.W. Chu, Raman spectroscopy of orthorhombic perovskitelike YMnO3 and LaMnO 3, Houston, Texas, 1998. https://doi.org/https://doi.org/10.1103/PhysRevB.57.2872.

[11] N. Jiang, S.M. Woodley, C.R.A. Catlow, X. Zhang, Applying a new interatomic potential for the modelling of hexagonal and orthorhombic YMnO3, J Mater Chem C Mater 3 (2015) 4787–4793. https://doi.org/10.1039/c4tc02759k.

[12] R. Maiti, S. Basu, D. Chakravorty, Synthesis of nanocrystalline YFeO3 and its magnetic properties, J Magn Magn Mater 321 (2009) 3274–3277. https://doi.org/10.1016/j.jmmm.2009.05.061.

[13] P.S.J. Bharadwaj, S. Kundu, V.S. Kollipara, K.B.R. Varma, Synergistic effect of trivalent (Gd3+, Sm3+) and high-valent (Ti4+) co-doping on antiferromagnetic YFeO3, RSC Adv 10 (2020) 22183–22195. https://doi.org/10.1039/d0ra02532a.

[14] O.S. Buassi-Monroy, C.C. Luhrs, A. Chávez-Chávez, C.R. Michel, Synthesis of crystalline YCoO3 perovskite via sol-gel method, Mater Lett 58 (2004) 716–718. https://doi.org/10.1016/j.matlet.2003.07.001.

[15] Y. Wei, H. Gui, Z. Zhao, J. Li, Y. Liu, S. Xin, X. Li, W. Xie, Structure and magnetic properties of the perovskite YCo0.5Fe0.5O3, AIP Adv 4 (2014). https://doi.org/10.1063/1.4904811.

[16] J.A. Alonso, J.L. García-Muñoz, M.T. Fernández-Díaz, M.A.G. Aranda, M.J. Martínez-Lope, M.T. Casais, Charge Disproportionation in RNiO3 Perovskites: Simultaneous Metal-Insulator and Structural Transition in YNiO3, Phys Rev Lett 82 (1999) 3871–3874. https://doi.org/10.1103/PhysRevLett.82.3871.

[17] C. Lu, C.H. Lee, T. Nishimura, A. Toriumi, Yttrium scandate thin film as alternative high-permittivity dielectric for germanium gate stack formation, Appl Phys Lett 107 (2015). https://doi.org/10.1063/1.4928749.

[18] J. Yue, N.F. Quackenbush, I. Laraib, H. Carfagno, S. Hameed, A. Prakash, L.R. Thoutam, J.M. Ablett, T.L. Lee, M. Greven, M.F. Doty, A. Janotti, B. Jalan, Electronic structure and small-hole polarons in YTiO3, Phys Rev Mater 4 (2020). https://doi.org/10.1103/PhysRevMaterials.4.112001.

[19] A.A. Tsvetkov, F.P. Mena, Y. Ren, I.S. Elfimov, P.H.M. van Loosdrecht, D. van der Marel, A.A. Nugroho, A.A. Menovsky, G.A. Sawatzky, Optical and magneto-optical study of orbital and spin ordering transitions in YVO3, Physica B Condens Matter 312–313 (2002) 783–784. https://doi.org/10.1016/S0921-4526(01)01494-6.

[20] A.N.L. Jara, J.F. Carvalho, A.F. Júnior, L.J.Q. Maia, R.C. Santana, On the optical and magnetic studies of YCrO3 perovskites, Physica B Condens Matter 546 (2018) 67–72. https://doi.org/10.1016/j.physb.2018.07.026.

[21] F. Zahrae Kassimi, H. Zaari, A. Benyoussef, A. El Kenz, Electronic and Magneto-Electric properties of YFeO3, J Magn Magn Mater 588 (2023). https://doi.org/10.1016/j.jmmm.2023.171414.

[22] T. Arima, Y. Tokura, J.B. Torrance, Variation of optical gaps in perovskite-type 3d transition-metal oxides, Phys Rev B 48 (1993) 17006–17009. https://doi.org/10.1103/PhysRevB.48.17006.
